# A comprehensive review of regulatory mechanisms of novel enterotoxin genes in *Staphylococcus aureus* from meat sources

**DOI:** 10.1080/07853890.2026.2628382

**Published:** 2026-05-04

**Authors:** Zhengping Guan, Yan Shi, Xiaolin Tian, Chengni Jin, Zhengjun Guan, Zheng Guan

**Affiliations:** a College of Food Science, Shanxi Normal University, Taiyuan, China; b Department of Life Sciences, Yuncheng University, Yuncheng, Shanxi, China; c State Key Laboratory of Vegetation and Environmental Change, Institute of Botany, Chinese Academy of Sciences, Beijing, China

**Keywords:** Meat-derived *Staphylococcus aureus*, novel enterotoxin genes (NEGs), egc gene cluster, transcriptional regulation, post-translational modifications, foodborne public health risk

## Abstract

**Background and aims:**

Meat-borne *Staphylococcus aureus* (*S. aureus*) remains a leading global foodborne pathogen harbouring novel enterotoxin genes (NEGs) encoding superantigenic toxins with conditionally enhanced pathogenicity, representing a critical food safety hazard. This review characterizes NEG features, pathogenic mechanisms, multi-layered regulatory networks, and identifies key research challenges.

**Methods:**

We systematically searched PubMed, Embase and the Web of Science Core Collection to synthesize molecular and multi-omics evidence (ChIP-seq, RNA-seq, CRISPR-Cas9) characterizing NEG classification, pathogenic mechanisms, multi-level regulatory networks (transcriptional, post-translational, environmental), and geographical distribution patterns in meat-derived *S. aureus*.

**Results:**

NEGs comprise superantigenic and tissue-targeting subgroups mediating pathogenicity *via* cytotoxicity, intestinal microenvironment disruption, and immune evasion. Regulation involves a complex network of Agr/σB/SarA/Rot-mediated transcriptional control, phosphorylation/lactylation modifications and environmental sensing, exhibiting marked geographical divergence. Current limitations include technical resolution constraints, insufficient physiological model fidelity and incomplete regulatory crosstalk elucidation.

**Conclusions:**

Future research should prioritize transcription factor interaction mechanisms, growth-toxin correlation prediction models and multi-omics-based network decipherment. This review provides a foundational framework for NEG research to inform food safety risk assessment and targeted contamination control strategies.

## 1. Introduction

*S. aureus*, first identified from human suppurative lesions [1], is a major zoonotic pathogen and leading contributor to global foodborne illnesses [2]. It colonizes both humans and livestock, with cross-species transmission occurring *via* animal contact and food contamination, and meat products (particularly poultry and pork) serve as key vehicles for human infection [3]. Recurrent *S. aureus*-associated food poisoning is primarily driven by heat-stable staphylococcal enterotoxins (SEs) [4,5], a burden exemplified in Europe where SEs-related outbreaks account for 30**–**40% of bacterial toxin-associated foodborne diseases [2]. This public health burden highlights the urgency of deciphering the regulation of SEs – especially novel enterotoxin genes (NEGs) – in meat-derived *S. aureus*, the core focus of this review.

Classical SEs (SEA–SEE) have been well characterized since 1960 [6], but NEGs such as *seg*, *sei* and *selL* were identified decades later *via* genomic mining [7,8]. Unlike classical SEs, NEGs exhibit unique pathogenic traits: they possess superantigen activity, strong MHC class II binding affinity, making them key drivers of severe foodborne outbreaks [5,9]. The expression of NEGs in meat production environments is further influenced by critical stressors. For instance, fever-like temperature upregulates agr and virulence genes, but *sek*/*seq* are repressed in co-culture [10]. Tetracycline at sub-MIC levels modulates the expression of several *S. aureus* virulence factors, including toxins and surface proteins [11]. Despite their clinical and food safety significance, NEG regulatory mechanisms in meat-derived *S. aureus* remain far less understood than classical SEs – with no systematic synthesis of transcriptional regulation, post-translational modifications (PTMs), and environmental stress-mediated modulation, representing a critical knowledge gap.

Addressing this gap requires a focused synthesis of NEG regulatory networks in meat-specific contexts. Unlike general *S. aureus* virulence research, this review targets the unique interplay between meat matrix characteristics (e.g. nutrient composition, processing stress) and NEG regulation – leveraging evidence from molecular biology (ChIP-seq, CRISPR-Cas9) and bioinformatics (RNA-seq) studies [12,13]. By integrating these multidimensional data, we can clarify how transcriptional factors (AgrA, SarA) and Rot (Repressor of toxin), PTMs (phosphorylation, glycosylation) and environmental cues (temperature, antibiotics) collectively modulate NEG expression, toxicity and persistence – providing a targeted solution to the dual threat of antibiotic resistance and NEG-mediated foodborne infections.

This review aims to systematically synthesize the multilevel regulatory mechanisms of NEGs in meat-derived *S. aureus*, with three core objectives: (1) decipher NEGs’ sequence characteristics, toxic effects, immune evasion mechanisms and expression patterns; (2) clarify the individual roles and cross-talk of transcriptional regulation, PTMs and environmental stressors in modulating NEG function; (3) elucidate how meat-specific contexts shape these regulatory networks. Ultimately, this work seeks to deepen understanding of *S. aureus* virulence evolution, support food safety risk assessment and early warning systems, thus strengthening the management of NEG-associated foodborne infections and safeguarding public health.

## 2. An overview of biological characteristics

### 2.1. *General overview of* S. aureus

*S. aureus*, a Gram-positive coccus belonging to the Micrococcaceae family, is widespread in both natural and host environments, demonstrating remarkable versatility and adaptability [13]. Individual cells of *S. aureus* are Gram-positive cocci with a diameter of 0.5–1 μm. After 24-hour incubation, they form circular, smooth and convex colonies measuring 2–4 mm in diameter – an observation that reflects the bacterium’s robust environmental fitness across a range of temperatures, pH levels and osmolarities [12,14]. Recognized as a major cause of both localized and systemic infections in humans and animals [15], *S. aureus* further emphasizes its clinical importance. In addition to infectious diseases, it is a prominent contributor to foodborne illnesses [16], posing a significant threat to public health through the production of various toxins, including SEs, leukocidins and exfoliative toxins [17,18].

### 2.2. Foodborne infections and their global significance

In recent years, foodborne illnesses caused by *S. aureus* have emerged as a global public health concern, with frequent outbreaks reported worldwide. These outbreaks are often linked to the production of SEs, which constitute a substantial proportion (30–40%) of confirmed bacterial toxin outbreaks in Europe [2,19–21].

### 2.3. Specific case studies and epidemiological insights

Meat-derived *S. aureus* and its enterotoxins are a major global cause of foodborne illness, with distinct epidemiological patterns, toxin gene profiles and severity correlations observed across regions. Below is a concise, structured summary of key case studies, surveillance technologies and regional trends.

#### 2.3.1. Epidemiological burden and representative cases in China

*S. aureus*-induced food poisoning is among the leading causes of bacterial foodborne incidents in China, imposing a substantial public health burden. The research confirmed its high incidence, with recent data further validating this trend [22–26]. Two key cases and molecular studies underscore critical characteristics: one is the 2019 municipal supermarket outbreak, where contaminated food products carrying classical enterotoxins (SEA–SEE) induced nausea, vomiting and diarrhoea. Retrospective analyses of this outbreak by Liu et al. [27–29], Zhao et al. [30] and Song et al. [31] confirmed that the involved *S. aureus* strains were resistant to penicillin and tetracycline, highlighting the synergistic risks posed by concurrent toxin production and antibiotic resistance. The second is the Hangzhou molecular survey, in which *S. aureus* strains associated with local food poisoning harboured the egc cluster but lacked classical enterotoxins (SEA–SEE) genes (Table 1). This underscores the need to expand surveillance beyond classical enterotoxins (SEA–SEE) to NEGs [30,62,63].

**Table 1.** Global distribution of meat-derived *S. aureus* and enterotoxin gene profiles (2009–2022).

Year	Region/country	Main source of meat-derived *S. aureus*	Sample number	Strain number of meat-derived *S. aureus*	Detection rate %	References
2009	Iowa, USA	Retail meat products	165	27	16.4	[32]
2010–2011	USA	Retail meat products	3520	982	27.9	[33]
2011	Thailand	Retail pork	10	3	25	[34]
2013	Egypt	Retail meat products	200	19	9.5	[35]
2013–2018	South Korea	Retail meat products	4264	777	18.2	[36]
2014	Tibet, China	Retail Yak meat	218	18	12.4	[37]
2015	Egypt	Retail Beef	225	58	25.78	[38]
2016	Chile	Retail Pork	155	88	56.8	[39]
2016	Denmark	Retail meat products	145	100	69	[40]
2016–2017	Italy	Retail meat products	500	72	14.4	[41]
2016–2017	Wuhan, China	Retail Pork	3067	518	16.9	[42]
2017	Punjab, India	Retail Raw meat	408	89	21.8	[43]
2017	Chennai, India	Retail Pork	100	20	20	[44]
2018	South Africa	Retail Pork	236	22	9.32	[45]
2018	Cambodia	Retail meat products	532	155	29.1	[46]
2018	Egypt	Retail Beef	100	16	16	[47]
2018	India	Retail Pork	120	92	76.67	[48]
2019	Thailand	Fermented pork sausage	36	22	60	[49]
2019	Turkey	Retail Pork and mutton	452	96	22.6	[50]
2019	South Africa	Slaughterhouse chicken	46	29	63	[51]
2020	Indonesia	Retail Chicken	60	35	58.3	[52]
2020–2022	Turkey	Retail Beef	100	6	6	[53]
2020–2022	China	Retail meat products	298	20	6.71	[54,55]
2021	Greece	meat products	160	22	13.8	[56]
2021	South Africa	Beef and its products	400	13	3.25	[57]
2022	Iraq	Retail Beef	50	32	64	[58]
2022	Nigeria	Retail Chicken	368	110	29.9	[59]
2022	Pakistan	salted fish	50	13	26	[60]
2022	Brazil	meat food	100	77	77.27	[61]

*Note:* Retail meat products primarily include fresh/chilled pork, beef, and chicken; fermented pork sausage (Thailand, 2019) and salted fish (Pakistan, 2022) are processed meat exceptions, specified to avoid category ambiguity. Defined as (number of *S. aureus*-positive strains/total number of meat samples tested) × 100%, calculated in Microsoft Excel 2019; samples with no bacterial growth (e.g. sterilized meat) were excluded from the denominator. Tibet, China (2014) samples are yak meat (a unique local meat source), and Brazil (2022) meat food includes beef jerky and minced pork, consistent with local consumption habits. All references used the same isolation method (TSA medium, 37 °C aerobic culture for 24–48 h) to ensure comparability of detection rates.

#### 2.3.2. Molecular detection as the technical basis for surveillance

Efficient epidemiological monitoring of meat-derived *S. aureus* depends on sensitive, specific tools. Guan et al. [64] developed a multiplex PCR (m-PCR) for 5 porkborne pathogens (including *S. aureus*), which showed high specificity, a low detection limit (<10 CFU/mL), and has been applied to pork samples [42,64].

#### 2.3.3. Global correlation between enterotoxin genes and outbreak severity

Multiple studies confirm a link between enterotoxin gene carriage, toxin protein levels, and outbreak severity across various food vehicles (Table 2). While most severe outbreaks historically associate with classical SEs, NEGs are increasingly recognized as contributors. Evidence from non-meat sources also highlights their potential; for example, in the 2005 Osaka (Japan) reconstituted milk outbreak, *seh*-positive *S. aureus* caused over 1,200 cases. More directly relevant to meat sources, the 2014 Tibet (China) yak meat outbreak involved *sel*-positive strains. However, the symptoms were mild, which correlated with low SEL protein expression and weak cytotoxicity [37]. This stark contrast between outbreaks underscores that for both classical and novel SEs, gene presence alone is insufficient to predict severity in meat-borne contexts.

**Table 2.** Association between enterotoxin gene carriage and foodborne outbreak severity.

Outbreak ID	Region/year	Contaminated food	Enterotoxin gene(s)	Strain characteristics	Outbreak scale	Severity indicators	Detection method	References
OSA-2005	Osaka, Japan /2005	Reconstituted milk	*seh*	Methicillin susceptible *S. aureus* (MSSA), penicillin-resistant	>1,200	Severe vomiting as the predominant symptom; a considerable proportion requiring hospitalization (Ministry of Health and Welfare, Osaka City, 2001)	PCR+ ELISA	[65]
TAI-2004	Taiwan, China /2004	Chinese sausage	*seg*, *sei*	MSSA	47	Diarrhoea as a universal symptom; a subset of patients presenting with fever.	PCR+Western blotting	[66]
WUH −2019	Wuhan, China /2019	Retail pork	*seg*, *sen*	MRSA (mecA⁺)	58	Bloody diarrhoea as a prominent severe symptom; a notable proportion of patients developing dehydration.	PCR+ LDH release assay	[42]
TIB-2014	Tibet, China /2014	Retail yak meat	*sel*	MSSA, ampicillin resistant, low SEL protein expression and weak cytotoxicity	18	61% with abdominal cramps, no hospitalization.	PCR+ CCK-8	[37]
SA-2017	South Africa /2017	Slaughterhouse chicken	*Sea, seb, sec, sed, eta, tst*	MRSA (mecA⁺)	/	tst-encoded TSST may induce toxic shock syndrome (TSS).	PCR	[51]

*Note:* The table demonstrates a direct correlation between specific enterotoxin genes (e.g. *seh*, *seg*/*sei*, *seg*/*sen*, *sel*) and the predominant clinical symptoms observed in outbreaks, ranging from severe vomiting and dehydration to diarrhoea (including bloody diarrhoea) and abdominal cramps. The severity of outbreaks is influenced not only by the presence of enterotoxin genes but also by key strain features, particularly methicillin resistance (MRSA vs. MSSA). The linkage between genetic detection and disease outcome was established using a suite of functional assays. LDH (Lactate Dehydrogenase) release assays quantified direct cellular cytotoxicity. CCK-8 (Cell Counting Kit-8) assays measured overall impacts on host cell viability and metabolic activity. Animal emetic models provided direct *in vivo* evidence of the toxin’s ability to induce vomiting, a hallmark symptom of staphylococcal food poisoning.

#### 2.3.4. Regional differences in toxin gene prevalence

Recent surveys reveal geographic variation in toxin gene distribution. In Eastern Turkey, 17.6% of meat-derived *S. aureus* isolates (*n* = 108) are MRSA, with 32.4% carrying *seb* and 18.5% carrying *sea* genes detected by PCR [67]. These strains show 66.7% penicillin resistance, linking antibiotic misuse to enterotoxin gene carriage. ST829 strains co-carrying mecA and *seb* exhibit high zoonotic risk, as these strains can express SEB toxin, a finding consistent with Eastern Turkey’s 25.8% SEB detection rate in retail beef and supporting the potential for zoonotic transmission of these *seb*-positive strains through contaminated meat products [67]. This highlights the need for targeted surveillance of *seb*-positive MRSA in Mediterranean meat supply chains.

Collectively, these epidemiological findings highlight three critical observations that motivate the subsequent focus on NEGs’ genetic and functional characteristics: (1) Regional variations in toxin gene prevalence reflect divergent evolutionary adaptation of meat-derived *S. aureus* to local food chains and storage conditions; (2) NEGs are consistently linked to severe foodborne outbreaks globally, yet their genetic distinction from classical SEA–SEE and mechanisms underlying their enhanced pathogenicity remain undefined; (3) Toxin gene carriage alone is insufficient to explain outbreak severity – protein-level expression and functional activity emphasize the need to characterize NEGs’ structural and genetic traits. To address these knowledge gaps, the following sections (3.1–3.2) define NEGs based on genetic criteria, classify them by sequence/functional features, and dissect their unique properties, thereby providing a molecular framework to interpret the epidemiological patterns observed above.

### 2.4. NEGs and regulatory mechanisms

#### 2.4.1. Definition and research gaps of NEGs

NEGs comprise all staphylococcal enterotoxin-like genes identified after SEA–SEE, unified by: (1) <50% amino acid identity to classical SEs, (2) distinct regulatory elements and (3) incomplete functional characterization in food matrices [7,68]. These genes expand the repertoire of enterotoxins produced by *S. aureus*, adding complexity to the regulation of toxin expression. While the regulatory mechanisms of classical staphylococcal enterotoxins (SEs) are incompletely characterized, understanding of NEGs regulation – particularly in meat-derived *S. aureus* – is far more limited. Current insights into SEs regulation highlight intricate crosstalk between environmental signals, transcriptional regulators and post-transcriptional modifications [69–72], yet critical knowledge gaps persist regarding how NEGs are regulated and their specific role in meat-associated foodborne illnesses. Elucidating these mechanisms is essential for developing targeted interventions to mitigate *S. aureus* contamination risks in the food chain.

#### 2.4.2. Temporal trends in NEGs research (2004–2024)

**Figure 1.**
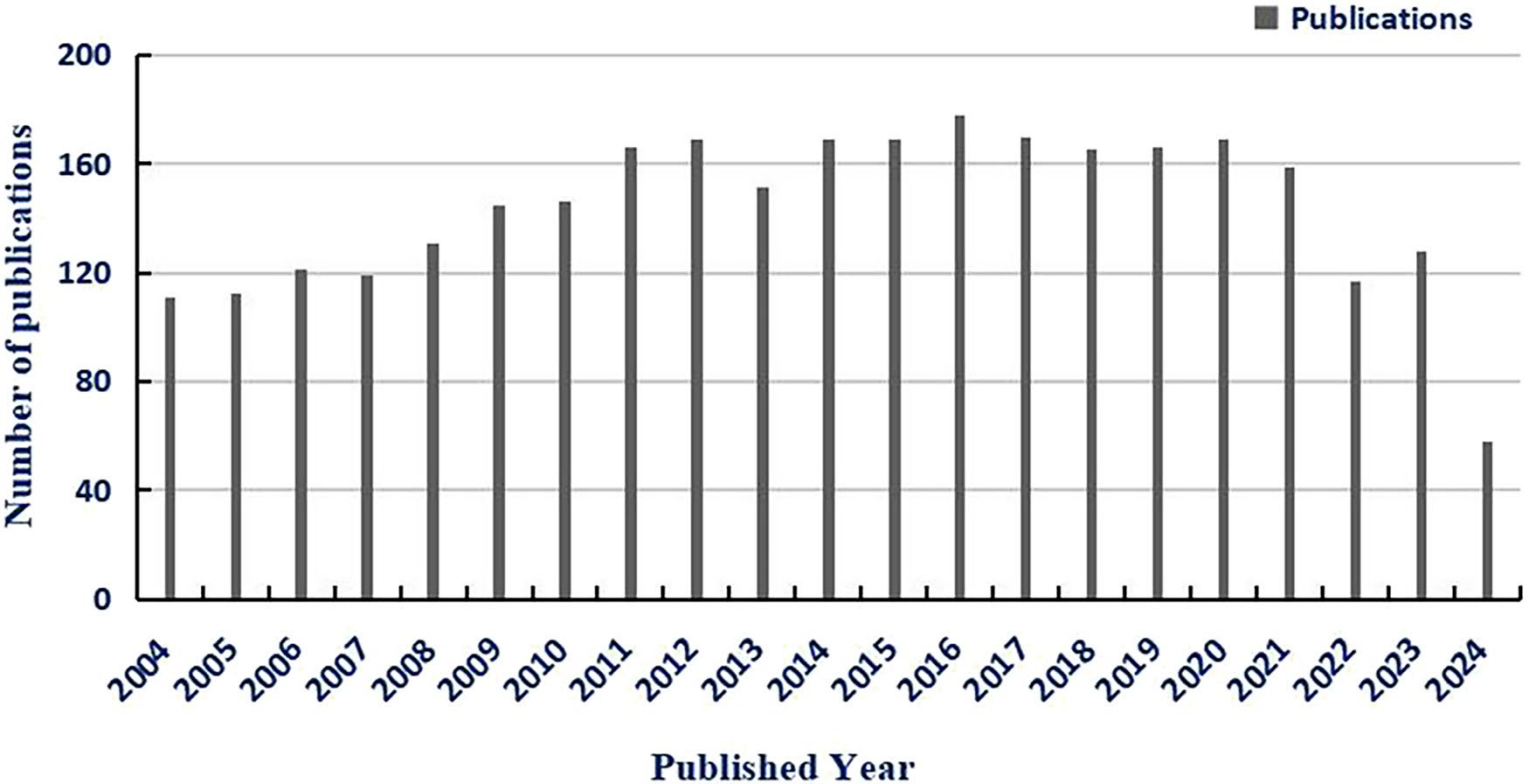
Temporal dynamics of publications on novel enterotoxin genes in *S. aureus* from meat sources (2004–2024). *Notes:* Publication data were retrieved from Web of Science Core Collection on (March 30, 2025) using the search query: TS=(‘novel enterotoxin*’ OR ‘egc cluster’ OR ‘SEG’ OR ‘SEI’ OR ‘SEM’ OR ‘SEN’ OR ‘SEO’ OR ‘SHE’ OR ‘SEK’ OR ‘SEL’) AND TS=(‘Staphylococcus aureus’) AND TS=(‘meat’ OR ‘food’ OR ‘retail’). Data were analyzed and visualized using Microsoft Excel 2019.

Figure 1 illustrates the temporal dynamics of scholarly publications focusing on NEGs in meat-derived *S. aureus* over the 20-year period from 2004 to 2024. A qualitative analysis of data sourced from the Web of Science Core Collection (WOSCC) reveals a clear non-linear growth trend in research output, reflecting the gradual recognition and maturation of NEGs as a critical food safety topic.

This trend can be divided into three distinct phases: (1) Initial phase (2004–2013): Low and sporadic publication output, primarily centred on the discovery of key NEGs (e.g. *seg*, *sei*) and preliminary prevalence surveys in meat matrices. (2) Growth phase (2014–2019): Steady increase in publication volume, driven by advancements in high-throughput sequencing technologies and heightened global attention to antibiotic-resistant foodborne pathogens. (3) Rapid expansion with late-stage retraction phase (2020–2024): Research activity first maintained a steady growth trend during 2020–2022, followed by a noticeable retraction in 2023–2024 – consistent with the negative annual growth rates of major research countries (Table 3). The initial growth was driven by the widespread application of multi-omics technologies in dissecting NEG regulatory mechanisms (Section 3.1.1) and the global emphasis on meat safety surveillance (Section 2.2). The subsequent retraction aligns with regional shifts in research priorities: for instance, early-leading countries (Germany, Italy) previously focused on classical NEGs (*seg*, *sei*) have shifted to interdisciplinary studies (Section 2.4.3), while emerging economies (India, Poland) show fluctuating output due to infrastructure development stages (Table 3). Additionally, the retraction may reflect the completion of core research on well-characterized NEGs (e.g. *seg*/*sei* regulatory pathways; Section 5.1.3) and a temporary lull before studies on understudied subtypes (e.g. *seq*, *sen*; Table 5) yield consistent publications. Notably, the growing publication volume underscores the escalating academic and public health interest in NEGs, with studies increasingly focusing on translational outcomes (e.g. rapid detection, targeted interventions).

**Table 3.** The annual growth rate of the number of published papers on novel enterotoxin of *S. aureus* in different countries (2020–2024).

Country	Growth rate in 2020 (%)	Growth rate in 2021 (%)	Growth rate in 2022 (%)	Growth rate in 2023 (%)	Growth rate in 2024 (%)
China	10.53	23.81	−23.08	75	−50
USA	7.69	−3.57	−40.74	6.25	−50
Brazil	0	−25	−66.67	−20	−76.47
Japan	44.44	−7.69	−66.67	25	−20
Germany	−27.27	−12.5	28.57	−11.11	−85.71
Italy	0	−63.64	25	−60	−100
South Korea	20	83.33	218.18	−51.43	−70.59
Poland	20	−33.33	175	−54.55	20
India	−28.57	120	−72.73	133.33	−85.71
France	0	33.33	−50	75	−71.43

*Notes:* To analyze global research trends on NEGs, we conducted a bibliometric analysis of publications indexed in Web of Science Core Collection (2020–2024). This analysis complements the systematic review of prevalence studies (Table 1) and outbreak investigations (Table 2), providing a comprehensive overview of the research landscape. The detailed search strategy and data processing methods are as follows.

This table includes 10 countries with complete annual publication growth rate data (2020–2024). Turkey, another leading country in NEGs research, is not included due to incomplete data for individual years. For the full list of 11 leading countries, see Section 2.4.4. The annual growth rate (GR) is calculated based on the difference between the number of papers in the current year and the preceding year, relative to the preceding year’s count. Employing an advanced growth rate formula, defined as Growth Rate = [(Year’s Publication Count - Preceding Year’s Publication Count)/Preceding Year’s Publication Count] × 100%, calculated in Microsoft Excel 2019. Derived from Web of Science Core Collection, with the search strategy: ‘novel enterotoxin* OR NEGs AND *S. aureus* AND meat OR pork OR beef*’. A negative growth rate indicates fewer publications in the current year than the previous year (e.g. Italy 2023: 60% decrease) but does not imply reduced research importance (may reflect focus shifts to other NEGs subtypes). These studies revealed distinct characteristics (Table 4).

**Table 4.** Comparative characteristics of classical enterotoxins SEA–SEE and NEGs in meat-derived *S. aureus.*

Enterotoxin gene category	Discovery period	Identification technology	Core functional features	Representative genes	Regulatory mechanisms	References
Classical (SEA –SEE)	Mid-twentieth century (1950s–1970s)	Ethanol precipitation, preparative isoelectric focusing, serological assays, gene cloning, nucleotide sequencing	Pyrogenic, superantigenic, emetic; heat/pepsin-resistant; bind MHC II and TCR to activate T cells.	*sea, seb, sec (SEC1–SEC3), sed, see*	Controlled by agr and sar global regulators; expressed in postexponential phase; repressed by catabolite repression.	[79–81]
Novel Enterotoxin Genes (NEGs)	1990s –2020s	Southern blot, gene cloning, nucleotide sequencing, Northern blot, emetic assay, murine T-cell proliferation assay, ELISA	Emetic activity; superantigenic; stimulates T-cell proliferation and production of IL-2 and IFN-γ.	*seg*, *sei*, *seh*, *sek*, *sem*, *sen*, *seo*	*seg* transcribed as 6.7-kb mRNA; steady-state mRNA peaks in logarithmic growth phase.	[7,30,65,82]

*Note:* Both classical SEs and NEGs share key pathogenic properties, including emetic activity and superantigenic function (stimulating T-cell proliferation and cytokine production). A defining characteristic of classical SEs is their notable resistance to heat and proteolytic digestion (e.g. pepsin), which contributes to their stability in contaminated food. The table explicitly notes that the functional validation of certain NEGs (e.g. SEG, SEI) required heterologous expression systems with an external promoter, as native genetic fragments failed to produce detectable toxin. This indicates that native expression levels of some NEGs in *S. aureus* under standard conditions may be low or tightly regulated.

**Table 5.** Summary of known novel enterotoxins in meat-derived *S. aureus.*

Novel enterotoxin	Gene name	Regulatory elements	Biological roles	References
Staphylococcal Enterotoxin G (SEG)	*seg*	6.7-kb mRNA; ribosome-binding site; peak steady-state mRNA in logarithmic growth; inducible *via* β-lactamase promoter	Superantigen; elicits emesis in rhesus monkeys; stimulates murine T-cell proliferation; induces IL-2 and IFN-γ production	[7–12,30]
Staphylococcal Enterotoxin I (SEI)	*sei*	The upstream region of *sei* lacks an obvious promoter sequence. The gene is not expressed from its native fragment without an external promoter.	SEI exhibits both emetic activity (induces vomiting in primates) and superantigenic activity (Induces murine T-cell proliferation and IL-2/IFN-γ secretion).	[7,42]
Staphylococcal Enterotoxin H (SEH)	*seh*	At the genetic level, the transcriptional regulators Rot and SarA are identified as inducers of SEH expression. The hld RNA is also upregulated in strong SEH. These elements are interlinked, as pH stabilization downregulates rot, sarA, and hld RNA .	SEH possesses emetic activity. It contributed to a mass food poisoning outbreak alongside SEA, with quantities as low as ∼3–19 ng/g detected in implicated food.	[65] [86]
Staphylococcal Enterotoxin T (SET)	*set*	The *set* gene is located on the plasmid pF5 (and related pF5-like plasmids), which also carries the *selj*, *ser* and *ses* genes.	*set* has weak emetic activity, as demonstrated in a primate model. Its specific role in pathogenesis is not yet fully established, but it belongs to the superantigen family.	[87,88]
Staphylococcal Enterotoxin M (SEM)	*sem*	*sem* is part of the enterotoxin gene cluster (egc) typically located on the genomic island vSaβ, a mobile genetic element (MGE). The egc cluster includes *seg*, *sei*, *sem*, *sen*, *seo*, and sometimes *seu*, and is often carried on MGEs such as pathogenicity islands (SaPIs).	*sem* encodes a staphylococcal enterotoxin (SE) that functions as a superantigen, capable of stimulating non-specific T-cell proliferation and inducing food poisoning symptoms. SEs are heat-stable, resistant to proteolytic digestion, and remain active in the gastrointestinal tract after ingestion, leading to staphylococcal foodborne illness.	[5,30,62,89]
Staphylococcal Enterotoxin Q (SEQ)	*seq*	SEQ is encoded by SaPI (Staphylococcal pathogenicity island), which is associated with the presence of prophage SGF. All MRSA strains harboured SGF prophage, suggesting a genetic linkage for seq expression.	SEQ functions as a staphylococcal enterotoxin capable of causing food poisoning. Its presence, along with sea and sek, indicates the potential of these strains to produce multiple enterotoxins, contributing to virulence and superantigen activity.	[90]
Staphylococcal Enterotoxin N (SEN)	*sen*	*sen* is located within the enterotoxin gene cluster (egc). The egc (including *seg*, *sei*, *sem*, *sen*, *seo*, *seu*) is typically carried together on the genomic island vSaβ, a mobile genetic element (MGE). This genomic context suggests its regulation and dissemination are linked to the egc cluster and vSaβ.	*sen* encodes a staphylococcal enterotoxin or enterotoxin-like (SEl) protein. Strains harbouring the egc cluster (including sen) can cause foodborne outbreaks, even without producing classical SEs. Its presence contributes to the enterotoxigenic potential and virulence profile of *S. aureus*.	[42,89]
Staphylococcal Enterotoxin L (SEL)	*sel*	A single nucleotide deletion in a poly(A) homopolymeric tract within the *sel* gene sequence was identified (in isolate Sa14-004). This deletion resulted in a premature stop codon, suggesting it affects gene expression/regulation.	The *sel* gene encodes a staphylococcal enterotoxin-like protein (SEI). The primary evidence for its biological role is its association with host adaptation, as phylogenetic clustering of *sel* sequences showed segregation between animal and human isolates.	[84,91]
Staphylococcal Enterotoxin R (SER)	*ser*	SEr are reported to be operons within the enterotoxin gene cluster of *S. aureus*, suggesting co-regulation at the genetic level.	The enterotoxin encoded by SEr exhibits strong thermostability, which may contribute to its persistence in contaminated foods after processing.	[92,93]
Staphylococcal Enterotoxin O (SEO)	*seo*	Direct evidence specific for *seo* regulation is not provided in the text. The literature states that *seo* is located within the egc (enterotoxin gene cluster), which is suggested to be an operon.	the *seo* gene, encodes enterotoxins (superantigens). These are emetic toxins and members of the pyrogenic toxin superantigen family.	[89,94]
Staphylococcal Enterotoxin J (SEJ)	*sej*	SEj are operons in the enterotoxin gene cluster in *S. aureus*. They are located close to each other genetically and show a synergistic relationship in expression and function.	SEj is the main enterotoxin gene in sheep and cattle with mastitis, suggesting its role in mastitis infection.	[48,92]
Staphylococ -cal Enterotoxin LL (SELL)	*sell*	The expression of the *sell* gene is positively regulated by the agr system. This is a direct regulatory relationship specific to sell.	The *sell* gene encodes a staphylococcal enterotoxin-like (SEI) protein. These proteins possess emetic activity but may differ in potency or function from classical enterotoxins.	[87,88,95]
Staphylococcal Enterotoxin K (SEK)	*sek*	*sek* is encoded by the SGF prophage. All MRSA strains in this study carried the SGF prophage type, which harbours *sea* and *sek* genes, suggesting prophage-mediated regulation and horizontal gene transfer.	SEK, as a staphylococcal enterotoxin, functions as a superantigen, modulating host immune response and contributing to bacterial persistence and potential foodborne intoxication. Its presence alongside other enterotoxins may enhance virulence.	[82,90]

*Notes:* The nature of evidence for regulatory elements varies. For some enterotoxins (e.g. SEH, SELL), direct experimental evidence (e.g. knockout studies, reporter assays) confirms the involvement of specific regulators (Rot, Agr). For others (e.g. SEJ, SEQ, SER), the regulatory information is often associated with their genetic context (e.g. presence in an operon or on a specific mobile genetic element) within meat-derived strains, as detailed in the provided references. The reported biological roles (e.g. emetic activity, superantigenicity) are established using heterogeneous experimental models (e.g. primate emesis models, murine T-cell proliferation assays). Caution is advised when directly comparing the potency or functional significance of different enterotoxins due to differences in assay systems, dosage, and model organisms.

#### 2.4.3. Geographical variations in NEGs research (2019–2024)

**Figure 2.**
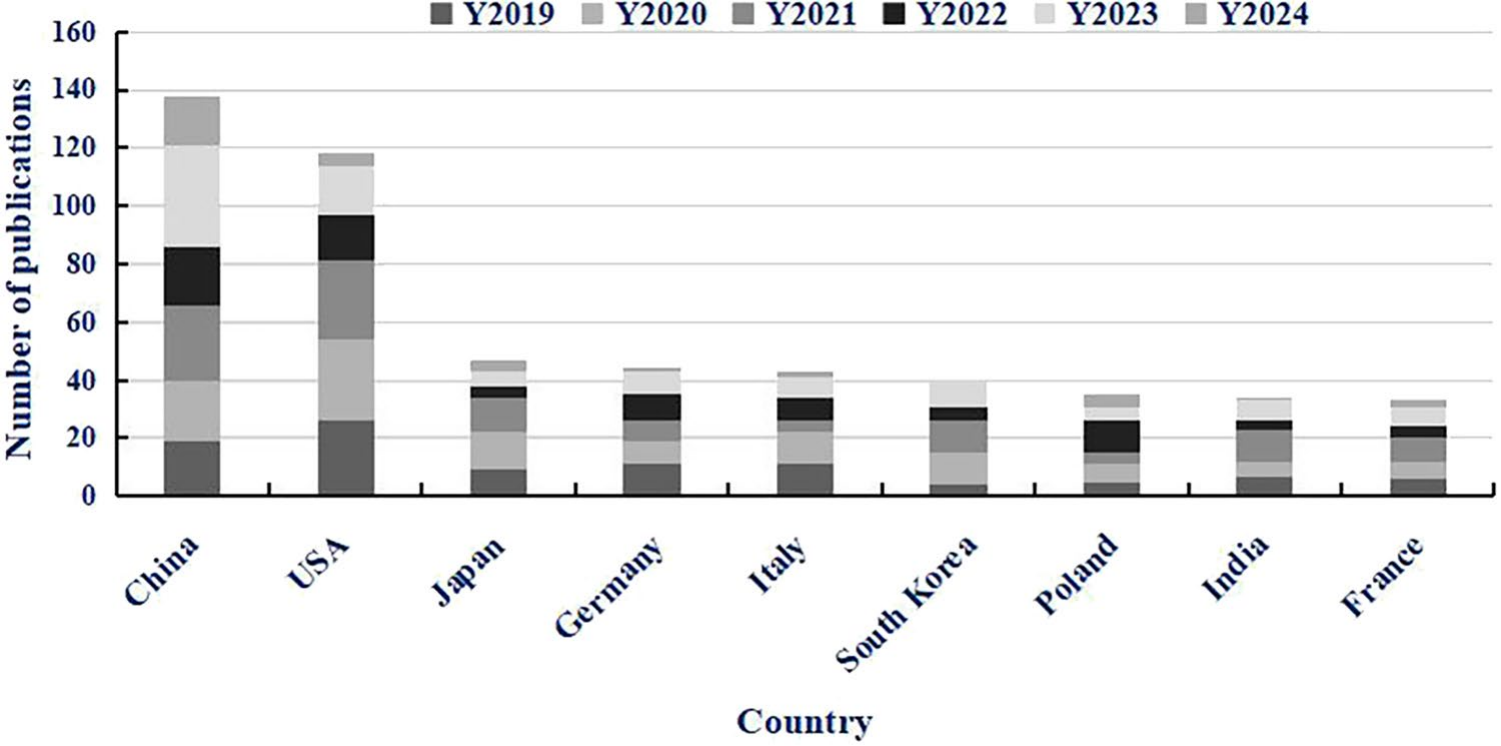
The total number of papers published on novel enterotoxins in different countries (2019–2024). *Notes:* Data were retrieved from Web of Science Core Collection (March 30, 2025). *seg*/*sei*/*seh* etc. represent representative novel enterotoxin genes (NEGs). This figure independently displays 9 core countries with top research output (2019–2024) and stable growth trends (China, USA, Japan, Germany, Italy, South Korea, Poland, India, France), which are key to supporting the regional research trend conclusion. The remaining 2 countries among the 11 leading countries (Brazil, Turkey) are included in the ‘Others’ category: Brazil is excluded from independent display due to extreme growth fluctuations (Table 3), and Turkey due to incomplete annual data and a research focus on non-core NEGs (*seb*/*sec*). For full data of 11 leading countries and 10 countries with complete growth rate data, see Section 2.4.4 and Table 3, respectively. Stacked bars represent annual publication counts (Y2019–Y2024). Data were analyzed and visualized using Microsoft Excel 2019.

A qualitative synthesis of WOSCC data (2019–2024) reveals distinct regional trends in NEGs research focused on meat-derived *S. aureus* (Figure 2; Table 3). European nations (e.g. Germany, Italy) showed initial research vitality, but their publication trends were characterized by significant fluctuations and subsequent negative growth in recent years: Germany exhibited a 28.57% growth in 2022 but a sharp decline of 85.71% in 2024, while Italy experienced consecutive negative growth from 2021 onward, with a 100% decrease in 2024.

In contrast, Asian countries (China, South Korea) and emerging economies (India, Poland) demonstrated phased growth with notable fluctuations rather than consistent expansion. China’s research output grew in 2020 (10.53%) and 2021 (23.81%) but declined in 2022 and 2024; South Korea achieved high growth rates in 2021 (83.33%) and 2022 (218.18%) but saw significant drops in 2023 and 2024; India had a dramatic surge in 2021 (120%) and 2023 (133.33%) but sharp declines in 2022 and 2024; Poland’s growth was intermittent, with positive growth in 2020 (20%), 2022 (175%) and 2024 (20%) offset by declines in 2021 and 2023. Detailed growth rate calculations are provided in Table 3.

#### 2.4.4. Regional research priorities in NEGs

Figure 2 further reveals geographically distinct research focuses for novel enterotoxins, with each region’s priorities closely tied to local toxin prevalence and food safety needs (Table 1). Specifically, China (Wuhan, Tibet) and South Korea – despite exhibiting fluctuating publication trends during 2019–2024 (Table 3) – prioritize research on the egc gene cluster (*seg*, *sei*, *sem*). This focus aligns with high local detection rates of related toxins: 16.9% of retail pork strains in Wuhan carry *seg* [42], while 12.4% of yak meat strains in Tibet are sel-positive [37]. In contrast, Turkey’s research centres on *seb* and *sec*, as 32.4% of local meat-derived MRSA strains harbour *seb* [67] – a pattern that reflects regional foodborne outbreak characteristics. For Germany and Italy, early research output was dominated by classical enterotoxins (SEA–SEE), but publication trends declined post-2021; this shift may be associated with the relatively mature research landscape of classical toxins and the reallocation of regional research resources towards other areas.

This geographical divergence clearly demonstrates that NEGs research priorities are primarily driven by local toxin prevalence and food consumption habits. Given these region-specific differences in research focus – coupled with the epidemiological significance of NEGs highlighted in Section 2.3 – it is imperative to first define and classify NEGs based on genetic and functional criteria (Sections 3.1–3.2). This foundational step will standardize research frameworks across regions, facilitating cross-study comparisons and addressing inconsistencies in how ‘novel enterotoxins’ are interpreted. It is worth noting that these trends are derived from available literature and may not fully reflect the latest ongoing research directions.

## 3. Classification and characteristics of NEGs

### 3.1. Discovery, definition and classification of NEGs

Building on the epidemiological evidence of region-specific toxin gene prevalence and NEGs’ association with severe meatborne outbreaks (Section 2.3), this section focuses on the discovery, definition and classification of NEGs. These genes – including *seg*, *sei* and *seh* – are distinct from classical enterotoxins in their genetic sequence, regulatory networks and pathogenicity. Clarifying their genetic identity is essential to explain why NEGs are more prevalent in certain meat supply chains and why they often cause more severe symptoms than classical toxins. Below, we define NEGs using criteria tailored to meat safety relevance and classify them based on sequence, structural and functional traits.

#### 3.1.1. Discovery process of NEGs

In recent years, the swift advancement of genomics and bioinformatics, particularly the pervasive utilization of high-throughput sequencing technology, has greatly facilitated in-depth investigations into the genome of *S. aureus* [24–26,73–75]. By scrutinizing the attributes of drug-resistant *S. aureus* isolated from retail food in Beijing and conducting whole-genome sequencing on these strains, researchers have successfully unveiled multiple NEGs [69,70]. High-throughput sequencing technology, characterized by its high throughput, precision and speed, has been instrumental in this discovery process [24–26,76]. Potential NEGs were pinpointed through comparative analysis with established enterotoxin gene sequences and bioinformatic examinations employing specialized tools, such as BLAST (Basic Local Alignment Search Tool) [77]. Subsequent experimental validations, encompassing gene knockout studies, expression analyses and toxicity assessments, have confirmed the functions and classifications of these putative genes [78]. This discovery journey underscores the pivotal role of integrating genomics and bioinformatics methodologies in deciphering the intricate nature of *S. aureus* genomes.

#### 3.1.2. Definition of NEGs

To eliminate ambiguity around ‘novelty’ and align with existing literature [7,65,68], NEGs are defined based on three complementary criteria tailored to meat safety relevance, rather than merely by discovery timeline: Discovery context refers to enterotoxin-encoding genes first identified *via* genomic technologies in the 1990s–2000s [7,8]. Amino acid homology relates to less than 50% identity with classical enterotoxins [68], which is a threshold widely accepted to delineate non-homologous toxin families [7]. Meat-derived research gaps refer to unresolved mechanisms of expression regulation, toxicity, or persistence in meat-specific contexts, which are understudied for NEGs but well-characterized for classical toxins.

A detailed comparison of core traits is provided in Table 4 to facilitate quick reference. Notably, genes like *seg* and *sei* [7], which are often categorized as ‘classical-like’ in broad classifications, remain classified as NEGs herein because they have distinct regulatory dependencies and relevance to meatborne outbreaks, and these traits differ substantially from these classical enterotoxins [10,8].

#### 3.1.3. Classification of NEG

Currently, the identified NEGs can be tentatively classified based on their sequence features, functional attributes and structural characteristics. One category of these NEGs, exemplified by SEQ, belongs to the superantigen family. Genes in this category encode proteins capable of non-specifically activating a large number of T cells, thereby triggering potent immune responses. Notably, the toxicity of most members in this subgroup often exceeds that of classical enterotoxins (SEA–SEE). However, it is important to emphasize that enhanced superantigen activity is not a universal trait across all NEGs – for instance, SET (Staphylococcal Enterotoxin T) exhibits only weak superantigen activity, which is consistent with its limited association with severe foodborne outbreaks (Table 5) [78,83–85]. Another category comprises NEGs that encode toxins specifically targeting gastrointestinal epithelial cells, potentially exhibiting tissue-specific targeting properties. The precise mechanisms of action underlying these toxins necessitate further elucidation. Furthermore, there exist NEGs encoding proteins that deviate structurally and functionally from conventional enterotoxins, such as those exhibiting unique enzymatic activities or signal transduction mechanisms. Additional research is required to investigate the prevalence and contribution of these NEGs to the virulence and pathogenicity of *S. aureus*. A limitation of the present classification system is the necessity for more comprehensive functional characterization of these novel genes, along with an examination of their distribution across diverse *S. aureus* strains.

**Figure 3.**
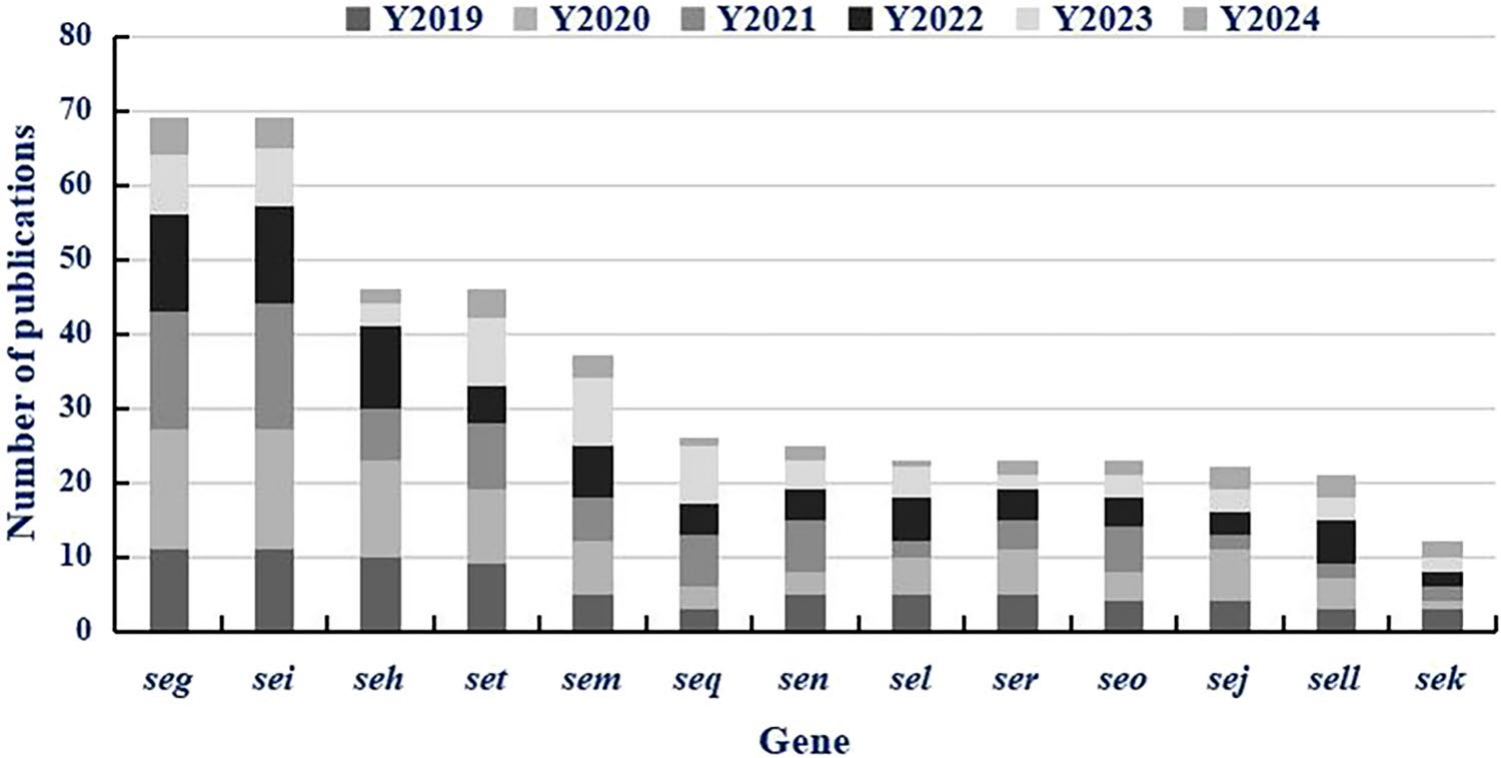
The total number of papers published on new enterotoxins in different Gene of *S. aureus* (2019–2024). *Notes:* Data were retrieved from Web of Science Core Collection on (March 30, 2025) using the search query: TS=(‘novel enterotoxin*’ OR ‘egc cluster’ OR ‘SEG’ OR ‘SEI’ OR ‘SEM’ OR ‘SEN’ OR ‘SEO’ OR ‘SHE’ OR ‘SEK’ OR ‘SEL’ OR ‘SEQ’ OR ‘SET’ OR ‘SER’ OR ‘SEJ’ OR ‘SELL’) AND TS=(‘Staphylococcus aureus’) AND TS=(‘meat’ OR ‘food’ OR ‘retail’). Thirteen NEGs are shown: seg, sei, seh, set, sem, seq, sen, sel, ser, seo, sej, sell, and sek. Stacked bars represent annual publication counts (Y2019–Y2024). Data were tabulated and visualized using Microsoft Excel 2019.

Figure 3 presents a comprehensive compilation of scholarly articles published on distinct genes associated with novel enterotoxins in *S. aureus*, encapsulating the research output over a six-year period from 2019 to 2024. Insights derived from the Web of Science database, utilizing a tailored search strategy focused on NEGs in meat-derived *S. aureus*, reveal a marked increase in the number of research papers during the six-year interval spanning from 2019 to 2024. Notably, the *seg*, *sei* and *seh* genes have garnered increased attention, with a pronounced surge in research publications pertaining to the *seg* gene in 2020 (Shown in Figure 3). The overall trend indicates a steady annual rise in interest in investigating NEGs, including *set*, *sem* and *seq*. This heightened research activity, bolstered by emerging policies, conference themes and funding trends, may mirror the escalating focus of scholars on food safety and disease prevention [53,96–99]. Researchers are actively delving into these NEGs, contributing to both the comprehension of their pathogenicity and the development of novel prevention and control strategies. Figure 3 illustrates that *seg*, *sei*, *seh* are the most studied novel genes (2019–2024), with *seg* publications surging 42% in 2020. This attention stems from three factors. (1) In the surveyed food samples, only 1.4% (2/139) of S. aureus strains carried both *seg* and *sei*, and their clinical role remains unclear [66]. *seh* caused Osaka’s 2005 reconstituted milk outbreak [65]; (2) Detection challenges lie in *seg* and *sei* being part of the *egc* cluster, which is easily missed by classical enterotoxins (SEA–SEE) detection methods [100]; (3) Environmental adaptability is evidenced by *seg* expression upregulated at 37–42 °C [101], making it a key target under climate warming. In contrast, *seq*, *sen* have lower research output, likely due to their lower prevalence and weaker association with severe outbreaks. This trend suggests future research should balance between high-risk genes and understudied genes to fill detection gaps.

### 3.2. Characteristics of NEGs

#### 3.2.1. Sequence and structural divergences

Classical enterotoxins (SEA–SEE) have long been extensively investigated, while the emergence of NEGs has been facilitated by advancements in molecular biology techniques. Compared to classical enterotoxins, NEGs exhibit distinct sequence and structural traits: Significant nucleotide sequence divergences, indicating divergent evolutionary trajectories or adaptive mechanisms [102,103]. Unique structural domains, such as additional glycosylation or phosphorylation sites, which may modulate stability, immunogenicity, or toxicity [37,104]. For detailed comparisons of homology and discovery context, refer to Section 3.1.2 and Table 4.

#### 3.2.2. Functional characteristics of NEGs

*S. aureus* is a prominent foodborne pathogen, with its secreted enterotoxins posing major threats to food safety and public health. It produces at least 22 distinct enterotoxin types, among which classical enterotoxins (SEA–SEE) have long been primary drivers of food poisoning – triggering vomiting and diarrhoea *via* T cell receptor (TCR)-mediated signaling [105]. The discovery of NEGs has expanded our understanding of enterotoxin diversity and identified new targets for antitoxin development, addressing critical gaps in foodborne disease control [100]. Below is a structured analysis of NEGs’ functional traits, focusing on mechanistic coherence.

##### 3.2.2.1. Structural basis of superantigenicity

NEGs exhibit superantigen activity through distinct structural domains that mediate specific interactions with TCR Vβ and MHC class II molecules. This is exemplified by Group V superantigens such as SEK, whose unique α3‑β8 loop extends the TCR binding interface by contacting the apical loop of the TCR Vβ FR4 region. Key residues within this loop form essential hydrogen bonds and van der Waals interactions that dictate Vβ specificity [106]. Similarly, in classical superantigens like SEA/SEIP, the specificity of SAG–TCR interaction is governed by critical residues, whose physicochemical properties modulate binding affinity within the SAG–MHC class II complex [107].

##### 3.2.2.2. Quantitative TCR activation & signaling

NEGs exhibit tissue-specific TCR binding and activate non- redundant signaling pathways, amplifying pathogenicity: (1) Staphylococcal enterotoxins (SEs), such as SEB, are thought to contribute to gastrointestinal symptoms by inducing the release of pro-inflammatory cytokines, activating intestinal T cells, and disrupting the epithelial barrier. However, the specific emetic mechanism of individual subtypes like SEH, as well as the role of TCR Vβ specificity and T cell enrichment in the gut-associated lymphoid tissue (GALT), requires further investigation [108]. (2) As superantigens, SEs bypass conventional antigen processing by binding directly to MHC class II molecules and specific Vβ regions of the T cell receptor. This leads to polyclonal T cell activation, proliferation, and a massive release of cytokines, thereby driving intestinal immunopathology. The detailed intracellular signaling cascades downstream of this interaction remain to be fully elucidated [109].

##### 3.2.2.3. Pathological consequences

Functional traits of novel enterotoxins (NEGs) lead to distinct pathological outcomes, as evidenced by protein-level data (Tables 5 and 6). (1) SEK acts as a superantigen, inducing the proliferation of human PBMCs and secretion of pro-inflammatory cytokines at 10 ng/mL [82]. *In vivo*, anti-SEK monoclonal antibodies raise murine survival during infection, confirming its role in severe outcomes such as diarrhoea [82]. (2) SEL exhibits emetic activity, inducing vomiting in the house musk shrew model at 500 μg, and stimulates rabbit splenocyte proliferation at 1.0 μg/well, supporting its contribution to food poisoning [5,8]. (3) SEH also shows emetic activity in the shrew model, though it requires higher doses than classical SEA/SEE [5]. (4) SEM, encoded within the egc cluster, induces emesis in Suncus murinus at 1000 μg and has been linked to gastrointestinal outbreaks [30]. These protein-level validations directly associate NEG expression with key virulence mechanisms, underscoring the need to move beyond genetic detection alone in risk assessment.

NEGs differ from classical enterotoxins *via* unique structural motifs, tissue-specific TCR binding, and enhanced pathogenicity in drug-resistant strains. Understanding these traits is critical for developing targeted inhibitors and improving food safety measures, ultimately mitigating NEG-associated foodborne diseases. The structural and functional traits of NEGs directly underpin their pathogenicity observed in epidemiological outbreaks (Section 2.3). Section 4 builds on these characteristics to dissect the molecular pathogenic mechanisms of NEGs, including their cytotoxic effects, disruption of the intestinal microenvironment and immune evasion strategies – linking genetic/functional traits to clinical outcomes.

**Table 6.** Protein-level functional validation of NEGs.

Novel enterotoxin	Encoding gene	Protein detection/functional assay	Key results	Biological/clinical significance	References
SEK	*sek*	Western blotting; Thermal stability assay; CCK-8.	(1) SEK induces 6-fold human PBMC proliferation at 10 ng/ml and pro-inflammatory cytokine secretion. (2) Anti-SEK mAbs (4G3/5G2/9H2) inhibit its activity; (3)4G3 + 5G2 combination raises mice survival to 80%.	Higher SEK protein levels correlate with severe diarrhoea in foodborne MRSA cases. Heat resistance explains residual toxicity after cooking.	[82]
SEL	*sel*	ELISA; IL-8 ELISA; Western blotting.	(1)500μg SEL induced emesis in 3/6 house musk shrews; (2) 1.0 μg/well SEL maximally stimulated rabbit splenocyte proliferation; (3) 100 μg/kg oral SEL caused no emesis in monkeys.	SEL is a novel superantigen with pathogenic potential, contributing to staphylococcal food poisoning; its functional traits inform mechanism research and risk assessment for related infections.	[5,8]
SEH	*seh*	ELISA; SDS-PAGE; Western blotting.	(1)500μg SEH induced emesis in 2/6 house musk shrews; (2) SEH required higher doses than SEA/SEE to elicit emetic responses.	SEH is a pathogenic staphylococcal enterotoxin with emetic activity, facilitating research on food poisoning mechanisms and related risk assessment.	[5]
SEM	sem	PCR;Whole Genome Sequencing (WGS)	(1)17 SEM-carrying *S. aureus* isolates caused gastrointestinal symptoms in 46 individuals; (2) 1000 μg SEM induced emesis in Suncus murinus.	SEM, an egc-encoded novel enterotoxin, contributes to staphylococcal food poisoning, informing outbreak surveillance and preventive strategies.	[30]

*Notes:* Functional confirmation of NEGs utilizes diverse protein-level methods, including immunodetection (Western blot/ELISA), stability tests, and bioassays (cell proliferation, cytokine release, *in vivo* emetic models). This integrated approach verifies toxin expression, stability, and biological activity. Experimental data directly connect NEGs to staphylococcal food poisoning traits. Validated functions include emesis induction (SEL, SEH, SEM), superantigenic T-cell activation (SEK, SEL), and correlation with clinical severity (e.g. SEK levels with diarrhoea). SEK’s heat stability explains post-cooking toxicity. Protein-level data establish the pathogenic potential of NEGs, crucial for public health risk evaluation of contaminated meat. Characterizing functional traits (e.g. emetic dose, heat resistance) informs toxicity mechanisms and supports countermeasure development (e.g. anti-SEK antibodies).

## 4. Pathogenic mechanisms of NEGs

### 4.1. Toxic action mechanisms of novel enterotoxins

*S. aureus*, a prevalent foodborne pathogen, produces enterotoxins that are primary contributors to food poisoning outbreaks. These small, heat-stable protein toxins retain their toxic activity even after heat treatment during food processing. Recently, novel enterotoxins have emerged as a focal point of scientific investigation, exhibiting intricate and distinctive toxic action mechanisms, which are primarily manifested in the following aspects.

#### 4.1.1. Cytotoxic effects and receptor binding specificity

*S. aureus*, a ubiquitous foodborne pathogen, produces heat-stable enterotoxins that resist food processing temperatures and remain toxic, making them major culprits in food poisoning. Among these, novel enterotoxins have emerged as a research focus due to their complex and unique mechanisms of action (Figure 4A, B). Novel enterotoxins exert their effects by binding to two categories of receptors present on intestinal epithelial cells, including TLR4 and SEG/SEI/SEK-specific receptors (Figure 4A). These receptors may be unique to novel enterotoxins or share similarities with those of other known enterotoxins, contributing to their specificity and potential cross-reactivity. For instance, SEK specifically binds TLR4 [82]. Receptor binding triggers two core intracellular signaling pathways. (1) The MAPK pathway (Figure 4A) functions through a cascade where MAPKKKs phosphorylate MAPKKs, which in turn activate ERK, p38 and JNK, and each of these triggers distinct downstream effects. ERK promotes cell proliferation, while p38 and JNK drive inflammatory responses. Specifically, p38 amplifies cytoskeletal rearrangement [110]; while SEH upregulates IL-8 secretion, an effect mediated by the p38 signaling cascade, SEK exerts a pro-apoptotic effect on intestinal cells through activation of the JNK pathway [5,27,29,66]. (2) The NF-κB pathway (Figure 4A) involves activation of the IKK complex, which mediates IκB phosphorylation and degradation, releasing NF-κB to translocate into the nucleus. This upregulates pro-inflammatory genes (e.g. TNF-α, IL-6) [110–114] and disrupts tight junctions [26,]. Activation of both pathways induces cytoskeletal rearrangement and increased membrane permeability, ultimately leading to cell death (Figure 4B). Apoptosis is mediated by caspase-3 and characterized by cell shrinkage; necrosis involves RIPK3, MLKL and RIPK1 [115,116] and leads to membrane rupture and release of inflammatory contents [27,31,117].

Figure 4.Mechanisms of action and pathological consequences induced by NEGs-encoded enterotoxins in *S. aureus* (A) Receptor binding and activation of MAPK/NF-κB pathways (B) Cytotoxic outcomes and intestinal barrier disruption.*Notes:* Novel enterotoxins are toxins encoded by novel enterotoxin genes (NEGs), e.g. SEG, SEI, SEK [82]. Receptors include TLR4 (broad-spectrum) and SEG/SEI/SEK-specific receptors on intestinal epithelial cells [82]. MAPK pathway regulates inflammation and cell death via ERK/p38/JNK [118]. NF-κB pathway drives pro-inflammatory cytokine production [118]. Apoptosis is caspase-3-dependent programmed cell death [119]. Necrosis is RIPK3/MLKL-dependent accidental cell death, characterized by membrane rupture and inflammatory content release [120]. Intestinal barrier consists of tight junctions and mucus layer maintaining integrity [27,29]. Intestinal barrier disruption was verified by reduced expression of tight junction proteins (e.g. ZO-1, occludin) and thinning of the mucus layer [27,29]. Diagram created using Microsoft Visio 2019.

**Figure:**
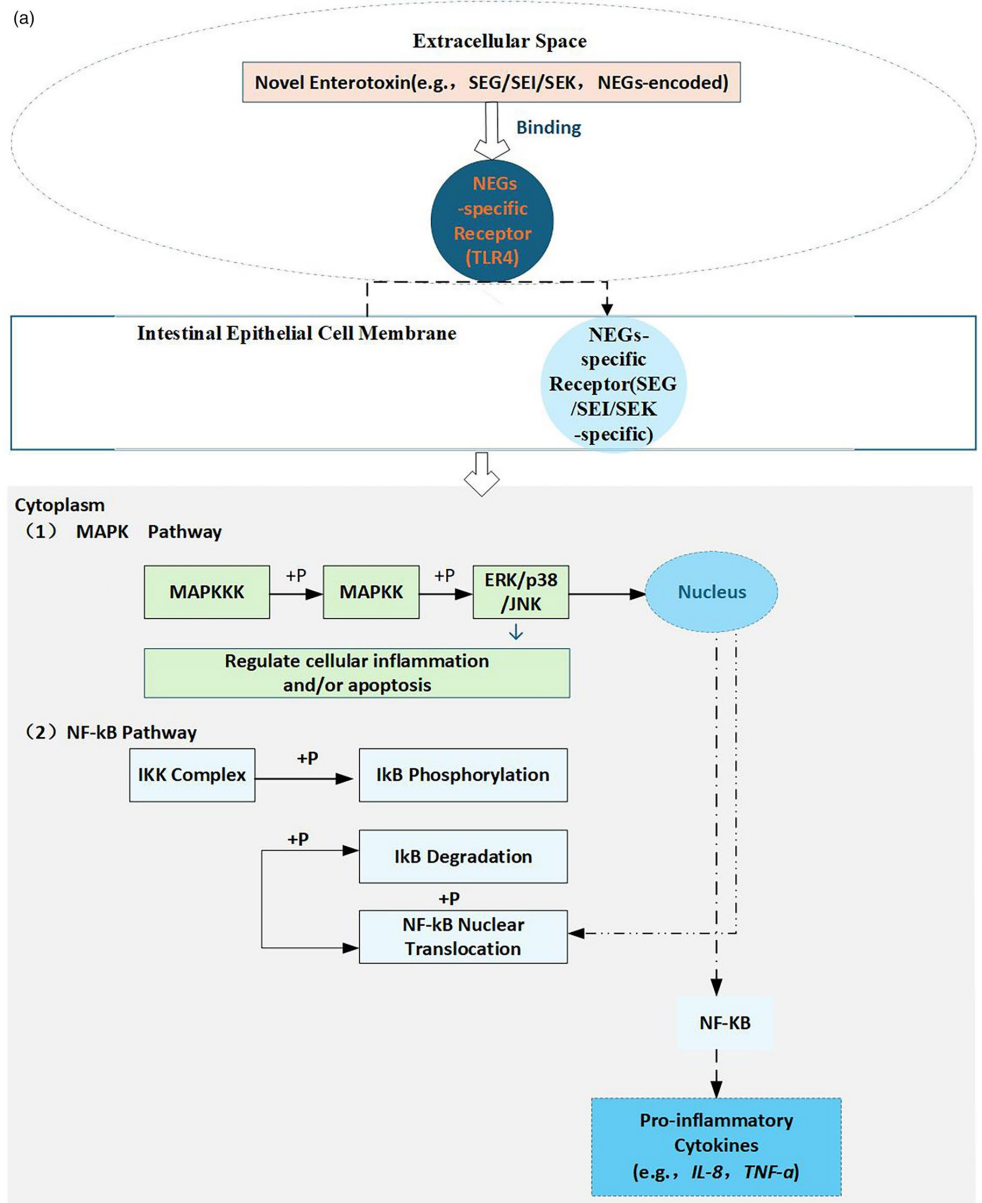

**Figure:**
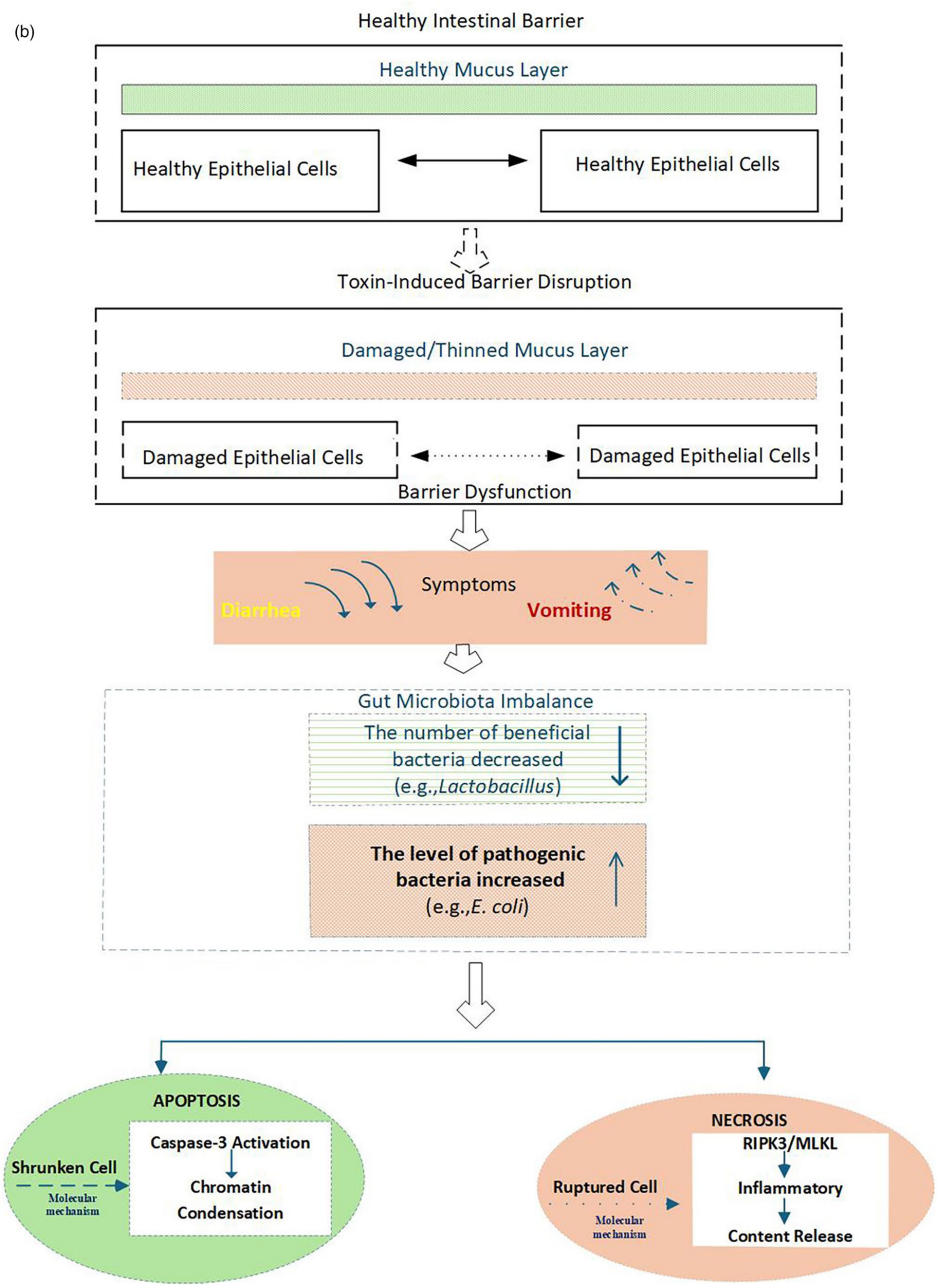

Cell death severely impairs intestinal barrier function, which relies on mechanical (tight junctions), chemical (mucus layer), immune and biological barriers. Disrupted tight junctions, thinned mucus layers and compromised immune/biological barriers impair water and electrolyte absorption, manifesting as acute gastroenteritis symptoms (diarrhoea, vomiting) [117]. Notably, such barrier disruption may also contribute to the onset and progression of inflammatory bowel diseases, highlighting the need for deeper mechanistic understanding to inform prevention and treatment strategies.

#### 4.1.2. Disruption of the intestinal microenvironment and activation of the MAPK and NF-κB pathways

Building on the MAPK and NF-κB pathway activation detailed in Section 4.1.1, novel enterotoxins primarily disrupt the intestinal microenvironment by disturbing the balance between beneficial and harmful bacteria. This process follows a clear sequential logic. The activated pathways first exacerbate intestinal barrier impairment (already described in 4.1.1), and the compromised barrier subsequently drives dysregulation of the intestinal microbiota (Figure 4A, B).

##### 4.1.2.1. Mechanism of pathway activation and microbial imbalance

The MAPK and NF-κB pathways, when activated by novel enterotoxins, exert distinct yet synergistic effects on intestinal microbiota homeostasis, primarily by modulating epithelial function and inflammatory responses.

MAPK pathway effects include the p38 subfamily mediating two key changes that alter microbial composition. Firstly, sustained p38 activation inhibits epithelial secretion of antimicrobial peptides (e.g. defensins), reducing populations of beneficial bacteria such as Lactobacillus [121]. Secondly, through the downregulation of H^+^-ATPase, p38 signaling elevates the intestinal luminal pH; this alkalization favours the expansion of pathogenic bacteria such as *E. coli* [122]. Additionally, activation of MAPK subfamilies like JNK contributes to epithelial cell apoptosis and necrosis (Figure 4B), destroying the physical scaffold that supports gut microbiota and further worsening microbial disorganization. The activation of the NF-κB pathway notably upregulates the expression of defensins. These antimicrobial peptides thereby selectively suppress commensal beneficial bacteria, including *Bifidobacterium* (Figure 4A). NF-κB also promotes the secretion of pro-inflammatory cytokines (e.g. IL-1β), which attract neutrophils but impair their phagocytic function. This forms a vicious cycle in which microbiota imbalance amplifies inflammation, and persistent inflammation further disrupts microbial homeostasis [123,124].

##### 4.1.2.2. Consequences of microenvironmental dysregulation

Intestinal microbial imbalance induced by novel enterotoxins has dual impacts. In the short term, overgrowth of harmful bacteria and heightened inflammatory responses synergistically disrupt gastrointestinal function, exacerbating the severity of food poisoning. In the long term, the imbalance compromises the microbiota’s core roles in food digestion, vitamin synthesis and immune modulation, triggering persistent health issues such as intestinal immune dysfunction and chronic inflammatory diseases [69,70,121,125–128].

#### 4.1.3. Modulation of neurotransmitters and interaction with the enteric nervous system

Novel enterotoxins may modulate neurotransmitter release from enterochromaffin cells, potentially contributing to vomiting, though direct evidence remains limited. This can lead to abnormal contractions or relaxations of intestinal smooth muscle. Enterotoxins may stimulate intestinal endocrine cells, such as enterochromaffin cells, which secrete neurotransmitters like serotonin. These neurotransmitters then bind to receptors on vagal sensory neurons, transmitting signals through the vagus nerve to specific neurons in the brainstem, which are pivotal in the vomiting reflex. Upon receiving these signals, the brain may elicit defensive responses, such as nausea and vomiting, to eliminate toxic substances from the body [108,129–131]. Furthermore, the enteric nervous system (ENS) plays a critical role in mediating the exacerbation of intestinal inflammation induced by psychological stress. Psychological stress can activate the ENS, further intensifying intestinal inflammation, indicating that the ENS acts as a relay station linking psychological stress and intestinal inflammation [123,128]. Novel enterotoxins may also impact other neurotransmitters, contributing to the symptoms of food poisoning. Understanding these neuroregulatory mechanisms offers a novel perspective for developing therapeutic strategies to alleviate discomfort caused by enterotoxins and exploring the gut-brain interactions underlying food poisoning symptoms.

### 4.2. Specific pathogenic mechanisms of novel enterotoxins

Within the scope of this review, novel enterotoxins (NEGs) encompass the expanding family of staphylococcal enterotoxins identified since the 1990s, including but not limited to SEG, SEI, SEH, SEK, SEM and SEN. As delineated in comparative analyses (see Table 4), NEGs share fundamental pathogenic properties with classical SEs, namely emetic potential and superantigenic activity. However, their distinctiveness may arise from differences in genetic regulation, temporal expression patterns and contextual functionality. Notably, the functional validation of certain NEGs (e.g. SEG, SEI) required heterologous expression systems, suggesting that their native expression and contribution to virulence in foodborne contexts might be uniquely regulated. This underscores the importance of investigating the specific pathogenic mechanisms and ecological roles of NEGs in meat-derived *S. aureus*.

#### 4.2.1. Immune evasion strategies

Novel enterotoxins produced by *S. aureus* possess high thermal stability and protease resistance, making them resistant to destruction even during common food processing or cooking conditions, which is a key factor contributing to their persistence in contaminated meat products [132,133]. *S. aureus* employs various strategies to interfere with the human immune system, particularly by secreting proteins that inhibit leukocyte function, thereby evading immune recognition and clearance. The bacterium produces a molecule known as chemotaxis inhibitory protein of *S. aureus* (CHIPS), which competitively binds to specific receptors on the surface of polymorphonuclear leukocytes (PMNs), such as C5aR and FPR receptors, thus preventing PMNs from recognizing formyl peptide signals released by bacteria [133]. Additionally, there are CHIPS-like molecules, such as formyl peptide receptor-like 1 inhibitory protein (FLIPr), which effectively block formyl peptide receptor (FPR) recognition of formyl peptides with even higher potency [134]. Furthermore, novel enterotoxins (e.g. SElX and SElY) have been shown to interact with CHIPS-like molecules to enhance PMN chemotaxis inhibition, thereby reinforcing immune evasion, a synergy that was recently validated in a meat-borne *S. aureus* isolate study [135] and extends the earlier findings on traditional CHIPS/FLIPr function [133,134]. Overall, *S. aureus* utilizes these secreted proteins to inhibit the natural response of the immune system, enabling the bacteria to evade immune clearance within the host and increase the duration and severity of infection [136]. This strategy allows novel enterotoxins to persistently exert toxic effects within the host, thereby elevating the risk and severity of infection, particularly in the context of food poisoning and related diseases.

#### 4.2.2. Enhanced toxicity

Structurally, novel enterotoxins are characterized by a superior ability to interact with key host immune receptors. They show stronger binding to Toll-like receptor 4 (TLR4) and MHC class II molecules than traditional enterotoxins and can also bind nucleotide-binding oligomerization domain 2. This broadened recognition profile enables them to trigger significant toxic responses at concentrations orders of magnitude lower than those required for SEA [137]. Furthermore, these enterotoxins act on a broader range of receptor types, allowing them to trigger toxic responses at lower concentrations. This increased affinity and broadened receptor spectrum may be attributed to the optimized molecular structure of novel enterotoxins, facilitating more effective interactions with host cells.

Novel enterotoxins produce synergistic effects by activating multiple signaling pathways, potentially leading to significantly enhanced clinical symptoms. They simultaneously activate signaling pathways related to inflammation, apoptosis and cell proliferation, resulting in more complex pathological processes in the body. This multi-pathway activation may lead to more severe diarrhoea, dehydration symptoms, and even shock, thereby increasing the difficulty and complexity of disease treatment. Additionally, studies have investigated the relationship between enterotoxins and intestinal pathology, further emphasizing the significance of these signaling pathways in disease pathogenesis. The pathogenic mechanisms underlying novel enterotoxins are intricate and multidimensional, encompassing cellular toxicity, disruption of the intestinal microenvironment, neuroendocrine pathway modulation, and immune evasion. A thorough elucidation of these mechanisms not only unveils the molecular underpinnings of severe food poisoning induced by novel enterotoxins but also establishes a robust theoretical framework for the development of effective prevention and control strategies in the future.

## 5. Advances in research on the regulatory mechanisms of NEGs

Gene expression regulation acts as a biological switch, dictating the activation or repression of genes, as well as the timing and location of their expression, thereby governing various life activities within an organism. This complex process entails multiple levels and mechanisms of regulation, extending from the transcription of DNA into mRNA to the translation of mRNA into proteins. It is vital for organisms to adapt to environmental changes, maintain internal homeostasis and facilitate essential life processes such as growth, development and reproduction.

Bacterial gene regulatory mechanisms are intricate and varied, encompassing three primary levels: transcriptional regulation, wherein gene transcription is modulated through the binding of RNA polymerase to promoters and the influence of transcription factors; translational regulation, occurring during the mRNA-to-protein translation process; and post-translational regulation, involving modifications or degradation of newly synthesized proteins. Among these, transcriptional regulation emerges as a primary mode of gene expression regulation in bacteria. In *S. aureus*, the Agr system functions as a crucial quorum sensing system, regulating the expression of enterotoxin genes by detecting bacterial density [138]. Furthermore, *S. aureus* utilizes additional mechanisms, such as small RNAs and two-component signal transduction systems, for the precise control of enterotoxin gene expression [139].

### 5.1. Transcriptional regulatory mechanisms

In the domain of food safety, *S. aureus* originating from meat sources, along with its novel enterotoxins, poses considerable threats to public health. Among the environmental factors influencing toxin expression in meat-derived *S. aureus*, low temperature plays a critical regulatory role. Notably, cold stress has been shown to reshape the global regulatory network in Staphylococcus aureus, potentially involving alternative sigma factors such as σB, which can subsequently influence the expression of virulence genes including enterotoxins. This environment-specific adaptive response may contribute to the unique pathogenic profile of food-borne isolates during chilled storage [140,141], and it represents a typical transcriptional regulatory mechanism of novel enterotoxins in meat-borne *S. aureus*. A thorough grasp of such transcriptional regulatory mechanisms governing these novel enterotoxins is crucial for the prevention and control of food poisoning, thereby ensuring food safety. Recent years have seen remarkable advancements in the identification and functional characterization of key transcription factors, driven by the rapid evolution of molecular biology techniques.

#### 5.1.1. Identification of key transcription factors

The Agr system in *S. aureus* comprises a sophisticated two-component regulatory system encompassing AgrA and AgrC. This system not only modulates biofilm formation and antibiotic resistance but also exerts a profound influence on the transcription of NEGs. The Agr system achieves quorum sensing (QS) *via* small autoinducing peptides (AIPs), allowing bacteria to orchestrate advantageous group behaviours at high cell densities [142–145]. Sigma factors, including σB, along with global regulatory proteins like SarA and Rot, are integral to the transcriptional regulation of enterotoxin genes. These transcription factors precisely recognize and bind to specific DNA sequences within the promoter regions of toxin genes, thereby regulating the recruitment and activity of RNA polymerase and exerting precise control over the expression levels of toxin genes [146–148].

Activation of the Agr system results in the upregulation of a range of secreted toxins and extracellular enzymes, aiding *S. aureus* in evading host defences, facilitating tissue invasion and acquiring additional carbon and energy from host organisms. This system is intimately associated with the virulence and infection capacity of *S. aureus*, and consequently, inhibiting the Agr system has been proposed as an anti-virulence strategy [149,150]. However, the intricate mechanisms underlying invasive and potentially fatal *S. aureus* infections, as well as the specific role of the Agr system in these infection processes, remain incompletely elucidated, underscoring the necessity for further investigation into this chemical signaling system. These transcription factors (Agr, σB, SarA) form a regulatory network that fine-tunes NEGs expression in response to meat-derived environmental cues (e.g. nutrient availability, pH), as discussed in the following sections.

#### 5.1.2. New breakthroughs in functional analysis

High-throughput sequencing technology and chromatin immunoprecipitation (ChIP-seq) have transformed the research landscape by allowing researchers to pinpoint transcription factor binding sites throughout the entire genome. The deployment of these technologies has further elucidated the direct interaction mechanisms between transcription factors, such as AgrA, and the promoter regions of NEGs in *S. aureus* [151]. Traditional gene knockout *via* homologous recombination has been used to validate AgrA function, whereby deletion of agrA in meat-derived *S. aureus* results in marked downregulation of toxin gene expression, which confirms the Agr system’s role in density-dependent NEGs regulation [152]. For more recent studies, CRISPR-Cas9 has been applied to generate precise agrA mutants, yielding consistent results [153]. These studies not only enhance our comprehension of transcriptional regulatory networks but also lay crucial groundwork for the development of innovative prevention and control strategies in the future.

#### 5.1.3. Transcriptional regulators and their direct target enterotoxin genes

**Figure 5.**
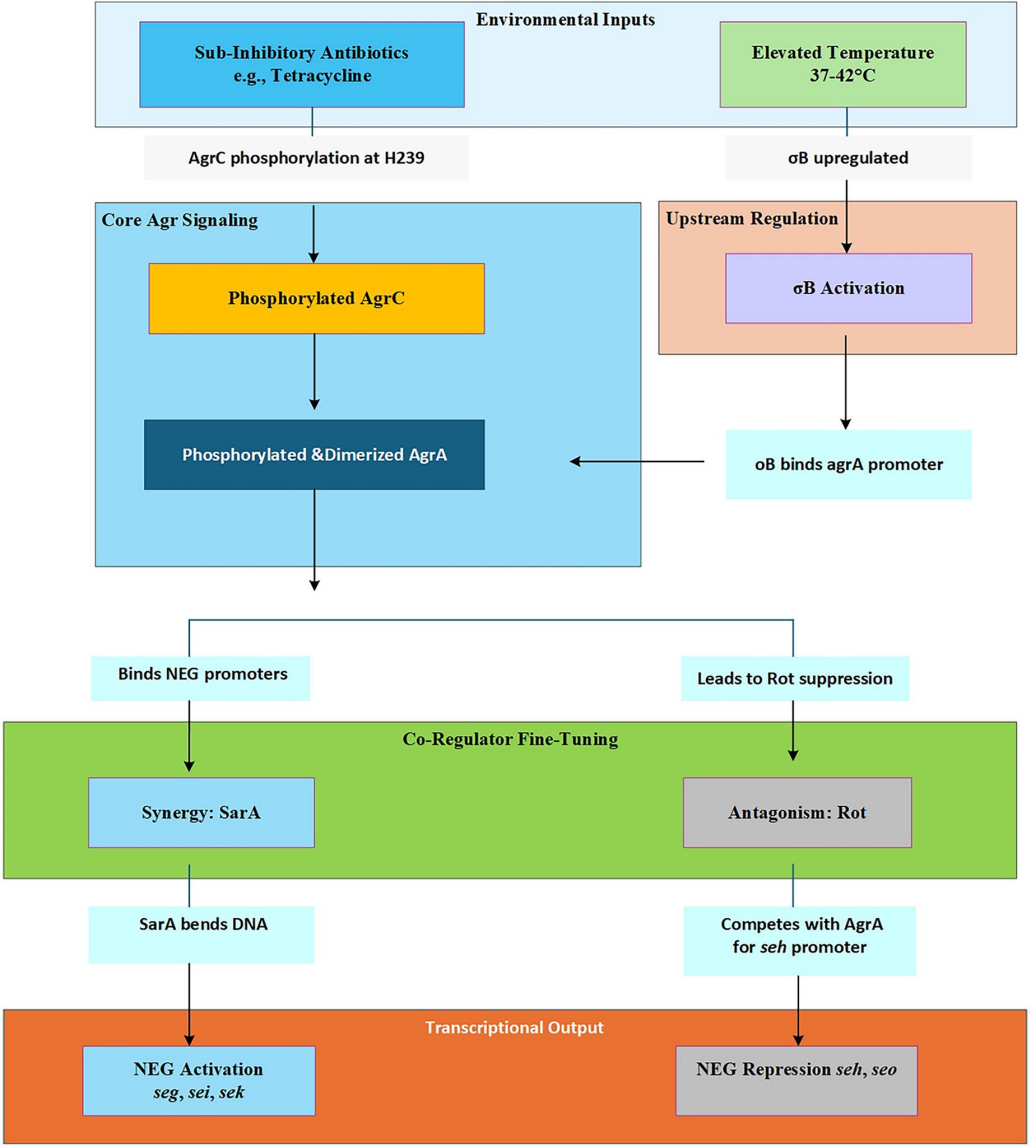
A hierarchical, evidence-driven model of the regulatory network governing NEGs expression in meat-derived *S. aureus*. *Notes:* The model delineates five functional layers through which environmental signals are integrated and transduced to differentially regulate NEGs’ transcription. Key external triggers initiate the cascade [154,155]. The sigma factor σB is activated by elevated temperature and directly upregulates the expression of the core regulator agrA [155,156]. Antibiotic stress and the translated AgrA protein drive the phosphorylation and dimerization of AgrA, enabling its binding to target promoters [156]. The output of the Agr system is precisely modulated by co-regulators; SarA acts synergistically with AgrA to potentiate toxin expression, while Rot acts antagonistically by competitive DNA binding [154,155,157]. This integrated network results in the concerted activation of high-risk NEGs (e.g. *seg*, *sei*, *sek*) and repression of other NEGs (e.g. *seh*, *seo*) [154,155]. Key experimental evidence supporting each step is indicated on the arrows, highlighting the mechanistic basis of this regulatory network. Diagram created using Microsoft Visio 2019.

The expression of novel enterotoxin genes (NEGs) in meat-derived *Staphylococcus aureus* is governed by a hierarchical and integrated transcriptional network, with the accessory gene regulator (Agr) quorum-sensing system serving as the central hub (Figure 5). This network transduces key environmental signals, such as elevated temperature and sub-inhibitory antibiotics, into differential transcriptional outputs for specific NEGs, through the coordinated action of upstream sigma factors, core Agr components and fine-tuning co-regulators.

##### 5.1.3.1. σB-mediated upstream activation of the Agr system

The alternative sigma factor σB acts as a critical upstream activator of the Agr system in response to environmental stress, particularly elevated temperature (Figure 5). Genetic studies have demonstrated that the production of enterotoxins encoded by the egc operon (including *seg*, *sei*, *sem*, *sen*, *seo*) is highest during early exponential growth and is dependent on σB [140]. This σB-dependent activation is proposed to occur through direct positive regulation of the agrA promoter, effectively priming the core quorum-sensing circuitry (Figure 5, Table 7). This layer of regulation integrates external stimuli into the NEG expression program.

**Table 7.** Direct regulatory relationships between transcriptional regulators and NEGs.

Transcriptional regulator	Target enterotoxin gene	Experimental technique	Regulatory effect	Key evidence	References
agr system	*seg, sei, sem, sen, seo*	Genetic analysis and AFLP (Amplified Fragment Length Polymorphism) clustering of 198 *S. aureus* strains to correlate toxin gene presence with agr phylogeny.	The enterotoxin gene cluster is positively associated with agr group IV. It shows a negative correlation with agr groups I and II.	The research demonstrated that the distribution of toxin genes is strongly linked to agr group specificity. The *seg*-*seo* cluster was found to be relevant specifically to agr group IV strains.	[158]
SacRS	*sel*	overall system analysis	Positive regulation	The SacRS system appears to have a positive impact on SEX expression. SEX belongs to the SEI group phylogenetically.	[159]
Rot	*seh*	Direct binding assay	Direct positive regulation	Rot directly binds to and activates the *seh* promoter, which is an exception to its typical role as a repressor.	[160]
σB	*ege operon (seg, sei, sem, sen, seo, seu)*	Genetic analysis (mutant studies)	Positive regulation	Production of ege-encoded enterotoxins is highest in early exponential growth and is dependent on σB.	[140]

*Note:* The regulatory relationships presented are supported by heterogeneous experimental evidence, ranging from direct molecular assays (e.g. binding studies for Rot) to genetic correlation analyses (e.g. AFLP clustering for agr). This highlights the varied levels of direct mechanistic validation for each regulator–enterotoxin pair. The identified regulatory interactions, such as the association of the *seg*-*seo* cluster with agr group IV or σB-dependent expression of the ege operon, are primarily derived from studies under specific laboratory or strain backgrounds. Their generality across diverse meat-derived *S. aureus* populations and under food-relevant conditions requires further confirmation.

##### 5.1.3.2. Agr-dependent regulation of NEG clusters

The phosphorylated and dimerized AgrA protein, the response regulator of the Agr system, directly binds to the promoters of target NEGs to drive their expression (Figure 5). A strong genetic association exists between the presence of the ***seg*-*seo*** enterotoxin gene cluster and strains belonging to agr group IV, highlighting a specific and positive link between the Agr system and this key NEG cluster [158, Table 7). This core signaling layer ensures population-density-coordinated expression of major toxin genes.

##### 5.1.3.3. Modulation by key co-regulators

The output of the Agr system is precisely fine-tuned by auxiliary regulators, notably SarA and Rot, which exert synergistic and antagonistic effects, respectively (Figure 5). SarA is known to synergize with AgrA to potentiate the expression of toxins like ***sei***. In contrast, the regulator Rot exhibits a complex role. While typically a repressor, Rot can directly bind to and activate the *seh* promoter, representing an exceptional case of positive regulation ([160], Table 7). Furthermore, Rot can act antagonistically by competing with AgrA for binding sites on other promoters, leading to repression of specific NEGs (Figure 5). This co-regulator layer adds a critical dimension of specificity and conditional responsiveness to the regulatory network.

##### 5.1.3.4. Integrated network output

The concerted action of this multi-layered network – integrating σB-mediated environmental sensing, Agr-dependent activation and SarA/Rot-mediated fine-tuning – results in the concerted transcriptional activation of high-risk NEGs (e.g. *seg*, *sei*, *sek*) and the context-dependent repression of others (e.g. *seh*, *seo*) in meat-derived *S. aureus* (Figure 5).

### 5.2. PTMs and their regulatory roles in novel enterotoxins

Post-translational modifications (PTMs) are crucial chemical alterations that occur after protein synthesis, modulating protein function, stability, localization and interactions. In *S. aureus*, PTMs play a pivotal role in regulating the activity of novel enterotoxins (NEGs), thereby influencing their pathogenicity in foodborne contexts. This section summarizes key PTMs – including phosphorylation, lactylation and hydroxymethylation – and their validated in modulating NEG function, with experimental evidence derived primarily from meat-derived *S. aureus* isolates (Table 8).

**Table 8.** Enterotoxin of post-translational modification (PTMs) and their functional validation.

PTM type	Gene/protein	Experimental technique	Key results	Biological significance	References
Phosphorylation	*Seb*, Enterotoxi-n genes	Phosphoproteomics (LC-MS/MS) and RT-qPCR	Eugenol significantly down-regulated the phosphorylation level of AgrA (fold change 0.52) and reduced the transcription of agrA and its downstream effector RNAIII, which controls enterotoxin genes like *seb.*	Inhibiting AgrA phosphorylation disrupts the QS system, leading to reduced expression of enterotoxin genes (e.g. *seb*), thereby attenuating virulence.	[69,70]
Ser/Thr phosphorylation	VraR	*In vitro* kinase assay with purified Stk1 and [γ-³³P]-ATP; Mass spectrometry (MS/MS) of tryptic peptides; Site-directed mutagenesis (Thr→Ala/Asp); Phos-tag SDS-PAGE; Electrophoretic mobility shift assay (EMSA)	VraR is phosphorylated by Stk1 at Thr106, Thr119, Thr175, and Thr178. Phosphorylation reduces DNA-binding to the vraRS promoter. A phosphomimetic (Asp) mutant fails to restore antibiotic resistance in a ΔvraR strain .	First evidence in *S. aureus* that a eukaryotic-like Ser/Thr kinase (Stk1) modulates a two-component response regulator (VraR). Phosphorylation negatively regulates VraR’s DNA-binding and antibiotic resistance functions, revealing cross-talk between major signaling systems.	[161]
Reversible phosphorylation (Ser/Threonine)	STK (SA1063) and STP (SA1062)	*In vitro* kinase assay with recombinant STK (rSTK) and myelin basic protein (MBP) using [γ-³²P]ATP; Thin-layer chromatography of acid-hydrolyzed phosphorylated proteins.	rSTK autophosphorylates and phosphorylates MBP; phosphorylation occurs preferentially with Mn²⁺ and targets threonine residues; rSTP dephosphorylates both autophosphorylated STK and phosphorylated MBP.	STK/STP-mediated reversible phosphorylation regulates cell wall integrity and antibiotic susceptibility in *S. aureus*; loss of STK increases sensitivity to β-lactam antibiotics, while STP deletion leads to thickened cell walls and lysostaphin resistance.	[162]
Lactylation of lysine residues	Lactylase genes	Techniques include LC-MS/MS for lactylome analysis, lactylase activity assays with purified enzymes and synthetic peptides, immunoblotting using Pan-αKla and αHlaK84la antibodies, and site-directed mutagenesis (K84R).	Lactylation at K84 of alpha-toxin is required for its full cytolytic activity. Deletion of lactylases SAPIG1173/SAPIG2573 or the K84R mutation reduces haemolysis, cytotoxicity, and virulence in mouse infection models.	This lactate-dependent PTM links host infection microenvironment (high lactate) to enhanced bacterial virulence *via* toxin activation, revealing a novel virulence regulation mechanism. Lactylases represent potential anti-virulence drug targets.	[72]
Hydroxymethylation of asparagine and glutamine	*Multiple proteins identified (e.g. Protein A, IsdA, IsdB, OMP7, etc.)*	Trypsin-shaving of live *S. aureus* cells followed by high-resolution LC-MS/MS (LTQ-Orbitrap XL/Velos) with CID fragmentation and Mascot database searching.	A mass shift of +30.0106 u was observed in 35 peptides from 15 proteins, corresponding to hydroxymethylation of Asn/Gln. Modification was specific to iron-poor (RPMI) conditions, not detected in iron-rich (TSB) medium.	Modification is enriched on surface/exposed proteins, suggesting a possible role in virulence modulation. It is a genuine PTM, not an artifact, and its status should be revised from ‘dubious’ to confirmed.	[163]

*Note:* Specific PTMs – phosphorylation, lactylation, and hydroxymethylation – directly modulate the activity of key virulence factors and regulators in *S. aureus*. For instance, phosphorylation of AgrA and VraR affects quorum sensing and antibiotic resistance, while lactylation of alpha-toxin enhances its cytolytic activity, linking metabolic conditions to pathogenicity. Each PTM is supported by orthogonal techniques, including mass spectrometry (LC-MS/MS), *in vitro* kinase assays, site-directed mutagenesis, and functional phenotyping (e.g. haemolysis, cytokine release). Hydroxymethylation of Asn/Gln occurs primarily under iron-poor conditions, and lactylation is driven by high lactate in the host microenvironment. This highlights how *S. aureus* adapts its proteome in response to environmental cues, fine-tuning virulence during infection. Enzymes mediating these PTMs, such as the lactylases SAPIG1173/SAPIG2573 and the kinase Stk1, emerge as plausible targets for anti-virulence strategies. Interfering with these modifications could attenuate toxin activity or restore antibiotic susceptibility without directly inhibiting bacterial growth.

#### 5.2.1. Phosphorylation

Phosphorylation is the most extensively studied PTM in *S. aureus* and acts as a reversible molecular switch to fine-tune enterotoxin activity, secretion and environmental adaptation. Phosphorylation of key regulatory proteins, such as AgrA, modulates quorum sensing and downstream expression of enterotoxin genes. Inhibition of AgrA phosphorylation by compounds like eugenol leads to reduced enterotoxin production, highlighting a direct link between phosphorylation and virulence regulation [69,70,164]. The serine/threonine kinase Stk1 and its cognate phosphatase Stp form an antagonistic pair that dynamically regulates phosphorylation states of virulence factors. Stk1 phosphorylates targets such as VraR, affecting DNA binding and antibiotic resistance [161], while Stp counteracts these effects to maintain cellular homeostasis [162,165].

#### 5.2.2. Emerging PTMs: lactylation, hydroxymethylation and their potential relevance

Beyond phosphorylation, other PTMs such as lactylation and hydroxymethylation have emerged as important regulators of *S. aureus* virulence, though their direct roles in modulating novel enterotoxins (NEGs) remain unexplored. Lactylation, induced under high-lactate conditions (e.g. within host niches), has been shown to potentiate toxins like α-haemolysin (Hla) by modifying lysine residues, thereby enhancing cytolytic activity and virulence [72]. Similarly, hydroxymethylation of asparagine/glutamine residues on surface proteins appears to be regulated by iron availability, suggesting a role in environmental adaptation [163]. While these modifications have not yet been directly linked to NEGs in meat-derived strains, they illustrate the broader PTM-mediated mechanisms through which *S. aureus* fine-tunes virulence factor activity in response to metabolic and environmental cues (e.g. nutrient composition, iron content in meat matrices). Investigating whether similar modifications occur on NEGs and influence their stability, secretion, or toxicity represents a promising future research direction.

### 5.3. Environmental factors and their impact on regulatory mechanisms

#### 5.3.1. The intricate interplay and pertinence of environmental factors

Environmental factors, encompassing global climate change and the pervasive use of antibiotics, exert profound effects on the gene expression and regulatory mechanisms of novel enterotoxins in *S. aureus*. These factors do not operate in isolation but rather interweave to form a complex regulatory network that modulates toxin production and pathogenicity.

#### 5.3.2. The influence of climate change

Climate change modulates the growth of meat-derived *S. aureus* and its expression of NEGs primarily through altering key environmental conditions, with temperature emerging as the most well-characterized and impactful regulatory factor. Below is a structured synthesis of evidence-based mechanisms, supporting experimental data, and unresolved gaps to clarify the logical link between temperature fluctuations and NEGs-related food safety risks.

##### 5.3.2.1. Temperature as a well-supported regulator of novel enterotoxin expression

Temperature governs NEGs expression *via* two interconnected, evidence-backed pathways – direct transcriptional regulation and indirect amplification *via* bacterial growth – both of which are validated by *in vitro* experiments and correlative epidemiological observations, though critical limitations remain. (1) Activation Elevated temperatures directly trigger NEGs transcription through the σB-Agr quorum-sensing axis. Specifically, increased thermal stress enhances the activity of the global regulator σB, which directly binds to the agrA promoter to upregulate its transcription [10]. (2) Temperature also modulates NEGs levels indirectly by accelerating *S. aureus* growth, which amplifies toxin accumulation in meat matrices. This pathway is quantified by a qPCR-based molecular predictive model established by Guan et al. [166] for artificially contaminated pork (temperature range: 7–30 °C), which exhibited strong fitting performance. A critical caveat is that all mechanistic evidence for temperature-driven NEGs regulation is derived from *in vitro* experiments, with no clinical epidemiological studies directly linking temperature-abused meat to increased severity of foodborne outbreaks.

##### 5.3.2.2. Host-microbe interaction links to NEGs

Climate change may indirectly exacerbate toxin-related risks by altering host-microbe interactions. For example, heat stress increases gut epithelial permeability in humans and animals, which could enhance the adherence of toxin-producing *S. aureus* to intestinal cells. However, no direct evidence links this process to the regulation of NEGs [167]. This represents a critical gap for future research.

#### 5.3.3. Public health ramifications and strategic approaches

Given the substantial impact of environmental factors on the regulatory mechanisms of NEGs in *S. aureus*, it is imperative to implement effective measures to optimize food storage conditions and promote judicious antibiotic use to curtail toxin production. These findings not only enhance our understanding of the biological attributes of *S. aureus* but also provide robust support for the formulation of more scientific and rational food safety policies [168,169].

## 6. Issues and challenges

### 6.1. Limitations of research techniques

In the endeavour to unravel the regulatory mechanisms of NEGs in meat-derived *S. aureus*, research techniques present notable limitations. Although cutting-edge technologies, such as high-throughput sequencing, transcriptomics and proteomics, offer substantial support, they are constrained by technical bottlenecks [170]. For instance, RNA-seq technology excels in portraying the gene expression landscape but falls short in precisely mapping the spatial configuration of regulatory elements and their complex interaction mechanisms with NEGs [171]. A prime example is the challenge in accurately pinpointing transcription factor binding sites, which hinders the interpretation of regulatory pathways. To circumvent this, it is recommended to enhance the application of computational biology tools, such as high-resolution techniques like Chromatin Immunoprecipitation with exonuclease treatment (ChIP-exo), to precisely locate these sites [172,173]. This should be complemented by advanced bioinformatics predictive models for a comprehensive analysis [129,166]. Furthermore, the field of epigenetic regulation in *S. aureus* warrants deeper exploration, as techniques like ChIP-seq and Methyl-seq require refinement, and data interpretation frameworks are still in their infancy. Interdisciplinary collaboration is suggested to develop species-specific detection methods and strengthen data standardization and quality control processes.

### 6.2. Emerging technologies and interdisciplinary collaboration

Emerging technologies, including single-cell sequencing and CRISPR-Cas genome editing, hold great promise in overcoming these limitations by providing enhanced resolution and precision [174,175]. Integrating these technologies with computational biology tools can facilitate a more profound understanding of the intricate regulatory mechanisms [176].

### 6.3. Complexity of regulatory mechanisms

The regulation of NEGs in *S. aureus* exhibits intricate, web-like complexity, involving environmental signals, host-microbe interactions and inter-strain heterogeneity [170,177]. This complexity requires multi-dimensional analyses, including transcriptional regulation, post-translational protein modification and environmental context dependency. For example, moderately elevated temperatures (37–40 °C) can enhance toxin production only when specific genes are activated and key proteins are phosphorylated. However, current *in vitro* models often fail to replicate the complex microenvironment of meat matrices or *in vivo* gut conditions, limiting the comprehensive understanding of regulatory networks. Thus, the development of more physiologically relevant models (e.g. meat-based microcosms) is urgently needed to dissect the interplay of these regulatory factors. The systems that control novel enterotoxins are exhibit high complexity driven by context-dependent factors. They depend on where the bacterium is (like in meat or the human gut), what the environment is like (hot/cold, with/without antibiotics), and even which strain of *S. aureus* it is. Because so many things affect it, scientists need new ways to study these systems to really understand how they work.

### 6.4. Limitations of experimental models and ethical considerations

The inadequacies of current experimental models in simulating the human physiological environment restrict the direct translation of research findings. Advanced models, such as humanized cell lines, mimic key human disease-associated phenotypes but lack the complex microbial community interactions present in meat or gut environments; intestinal organoid models replicate intestinal structure but require long-term culture and high costs; microphysiological systems (MPS) overcome the species-specific limitations of animal models but face challenges in scaling to high-throughput experiments [178].

## 7. Prospects

Future research on NEGs in meat-derived *S. aureus* will prioritize addressing critical knowledge gaps identified in this review, with a focus on mechanistic depth, translational applicability and food safety relevance. All directions build on existing findings to ensure logical continuity and actionable goals.

### 7.1. Construction of growth-toxin correlation prediction models

Building on the qPCR-based molecular growth model of *S. aureus* in pork – which quantified growth dynamics (lag time [LT], specific growth rate [SGR]) across 7–30 °C but lacked integration with NEGs expression [166] – this direction fills the gap between microbial quantity and toxin risk (Section 8.1). Specific Actionable Goals: (1) Validate correlations between *S. aureus* growth phases (lag/exponential/stationary) and transcriptional levels of high-risk NEGs in representative meat matrices using qPCR and ELISA [5,42,66]. (2) Integrate key environmental variables to simulate real-world storage conditions [146,166]. (3) Apply machine learning algorithms to integrate growth parameters, environmental cues and NEGs expression data, developing a user-friendly growth-toxin co-prediction tool [54]. This tool will enable real-time risk assessment of toxin production in meat supply chains, reducing over-reliance on post-hoc toxin detection.

### 7.2. Deciphering regulatory networks via multi-omics and gene editing

Section 5.1.3.3 identified SarA as a synergistic co-regulator that enhances AgrA-mediated *sei* expression, but the direct molecular interaction between AgrA and SarA remains uncharacterized. Specific Actionable Goals: (1) Use ChIP-seq to map overlapping binding sites of AgrA and SarA in the promoter regions of *sei* and *sem* in meat-derived *S. aureus*. Peak calling will identify conserved motifs co-bound by both regulators [10,42,58]. (2) Validate protein-protein interactions between AgrA and SarA using co-immunoprecipitation (Co-IP) and surface plasmon resonance (SPR) to quantify binding affinity, clarifying whether they form a complex to regulate NEGs [82,143]. (3) Generate AgrA/SarA double knockout strains *via* CRISPR-Cas9 to assess their synergistic contribution to NEGs expression. Conduct *in vitro* assays in meat matrix simulations to measure *sei*/*sem* transcription relative to single knockouts and wild-type strains [151,166].

### 7.3. Ethical, legal and social implications (ELSI)

Builds on findings that temperature (37–42 °C) and sub-inhibitory tetracycline regulate NEGs *via* σB-Agr and AgrC-AgrA pathways (Sections 5.3.2.1 and 5.3.3), but the global regulatory network under these stressors remains unclear [10,11]. Specific Actionable Goals: (1) Apply transcriptomics (RNA-seq) and phosphoproteomics (LC-MS/MS) to meat-derived *S. aureus* exposed to temperature stress or sub-inhibitory tetracycline. Identify differentially expressed genes/phosphoproteins involved in NEGs regulation [76,179]. (2) Integrate metabolomics data to link metabolic changes to NEGs expression under stress. Validate key regulators *via* CRISPR-Cas9 knockout, measuring *seg*/*sei*/*sek* transcription and toxin production [151,166]. (3) Develop a comprehensive regulatory network map that connects environmental stressors, upstream regulators, PTMs and NEGs expression – providing a systems-level understanding of toxin regulation in meat-specific contexts [42,54,55,170].

## 8. Conclusion and future directions

### 8.1. Key findings

This review clarifies the regulatory mechanisms of NEGs in meat-derived *S. aureus* and their implications for food safety. Core findings include the following.

(1) Multilevel regulatory networks of NEGs involve transcriptional regulation dominated by the Agr quorum-sensing system (AgrA/AgrC) and global regulators (σB, SarA) directly bind to specific regions of NEGs promoters to modulate expression [146,152]. PTMs further fine-tune toxin stability and toxicity, these traits distinguish NEGs from classical SEA–SEE [82,179]. (2) Environmental drivers play a critical role. Elevated temperatures (37–42 °C) upregulate NEG expression *via* σB-mediated Agr activation [10,69,70], while sub-inhibitory tetracycline induces *sek* expression [11,58], highlighting climate change and antibiotic misuse as key risk factors. (3) Insights into NEGs regulation have enabled the development of sensitive detection technologies (e.g. CRISPR-based sensors) and targeted interventions (e.g. Agr inhibitors), where early studies laid the foundation [100,153] and recent advances have enhanced their applicability [69,70,135]. (4) the integration of epidemiological data and protein-level evidence strengthens the public health relevance of NEGs, as they link genetic carriage to outbreak severity and toxic activity, which is critical for developing targeted food safety surveillance. (5) the experimental data on PTMs and direct transcriptional regulation clarify the mechanistic links between molecular modifications and regulators and enterotoxin activity, addressing the knowledge gaps in translational and transcriptional control of NEGs.

### 8.2. Future directions

To address remaining knowledge gaps and advance food safety, the following directions should be pursued. One direction is methodological innovations, which involve deploying multi-omics integration to dissect cross-talk between transcriptional regulators and PTMs; using CRISPR-Cas9-based high-throughput screening to validate NEGs regulatory elements [151]. Another is targeted regulatory studies, Utratna et al. [148] initially reported that the global regulator σB is involved in the cold stress response of *S. aureus*. Building on this work, Chen et al. [12] further confirmed that under low-temperature conditions (4 °C), σB directly binds to the promoter region of *seg* (a key NEGs) to suppress its expression. This study thereby uncovers the σB-mediated cold stress adaptation mechanism of NEGs, clarifying how *S. aureus* modulates enterotoxin production in chilled meat environments. A third is surveillance and intervention optimization, which involves developing portable, rapid detection devices (e.g. Lab-on-a-Chip) for on-site NEGs screening in meat markets [172], and establishing international collaborative networks to share NEGs sequence data and standardize detection protocols [168]. A fourth is physiological model validation, which involves using human intestinal organoid models to simulate NEGs-induced barrier damage, bridging the gap between *in vitro* findings and clinical manifestations (e.g. diarrhoea) [135,174].

## Data Availability

Data sharing is not applicable to this article as no new data were created or analyzed in this review.

## Author notes

Zhengping Guan (1975), Corresponding and First Author Introduction. She is a female researcher hailing from Hequ, Shanxi, China. She holds a PhD and currently serves as an Associate Professor. Her research interests span a wide range of topics, including meat safety and quality control, food storage and preservation, food quality and safety management, food nutrition and health, as well as the development of functional foods.
